# Transition from Light-Induced Phase Reconstruction to Halide Segregation in CsPbBr_3–x_I_*x*_ Nanocrystal Thin Films

**DOI:** 10.1021/acsami.4c19234

**Published:** 2025-02-20

**Authors:** Thiago Rodrigues da Cunha, Diego Lourençoni Ferreira, Letícia Ferreira Magalhães, Thaís Adriany de Souza Carvalho, Gabriel Fabrício de Souza, Jefferson Bettini, Angelo Danilo Faceto, Cleber Renato Mendonça, Leonardo de Boni, Marco Antônio Schiavon, Marcelo Gonçalves Vivas

**Affiliations:** † Laboratório de Espectroscopia Óptica e Fotônica, Universidade Federal de Alfenas, 37715-400 Poços de Caldas, MG, Brazil; ‡ Grupo de Pesquisa em Química de Materiais, Universidade Federal de São João del-Rei, 36301-160 São João del-Rei, MG, Brazil; § Laboratório Nacional de Nanotecnologia, Centro Nacional de Pesquisa em Energia e Materiais, 13083-970 Campinas, São Paulo, Brazil; ∥ Instituto de Ciências Agrárias, Universidade Federal dos Vales do Jequitinhonha e Mucuri, MGT Highway 367 – Km 583, no. 5.000. Alto da Jacuba, Diamantina-MG, 39100-000, Brazil; ⊥ Instituto de Física de São Carlos, Universidade de São Paulo, São Carlos, SP 13566-590, Brazil

**Keywords:** CsPbBr_3-x_I_*x*_ nanocrystal, perovskite nanomaterials, phase reconstruction, phase segregation, hyperspectral fluorescence microspectroscopy, Monte Carlo approach

## Abstract

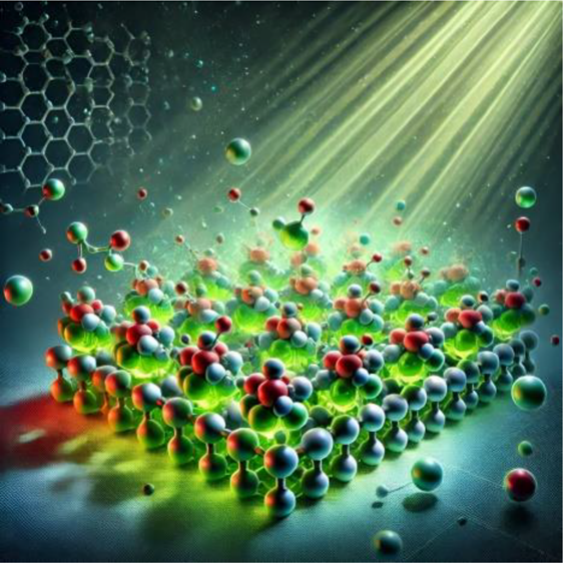

Inorganic metal-halide perovskite materials pave the way for many applications ranging from optoelectronics to quantum information due to their low cost, high photoluminescence and energy conversion efficiencies. However, light-induced bandgap instability due to ion migration in mixed-halide perovskites remains a significant challenge to the efficiency of optoelectronic devices. Thus, we combined hyperspectral fluorescence microspectroscopy and computational methods to understand the underlying transition mechanism between phase reconstruction and segregation in CsPbBr_3-x_I_*x*_ (0 < *x* < 3) nanocrystal thin films. Our outcomes have shown that samples with x = 1.0 and x = 1.5 exhibit halide migration, favoring Br enrichment locally. In this case, an interplay between photo and thermal activation promotes the expulsion of I^–^ from the perovskite lattice and generates a reconstruction of Br-rich domains, forming the CsPbBr_3_ phase. Thus, thermodynamic parameters such as the halide activation energy and phase reconstruction diffusibility were obtained by combining the kinetic parameters from linear unmixing data and Fick’s second law. Moreover, we observed that the Br–I interdiffusion followed an Arrhenius-like behavior over laser-induced temperature increase. On the other hand, for samples with x = 2.0, phase segregation occurred due to the larger CsPbBrI_2_ nanocrystal size, iodine content and the high laser intensity employed. These three combined effects modify transport and recombination due to the reduction of charge carrier diffusion length (L_D_ = 10.2 nm) and bandgap. Thus, iodide ions diffuse from the nanocrystal surface to the core forming a “type-II heterostructure”, promoting a red shift in the fluorescence spectrum, which is characteristic of phase segregation. Furthermore, real-time dark recovery of light-induced halide segregation is reported for CsPbBrI_2_ nanocrystal thin films. Finally, the possible halide migration mechanism and physical origins of the transition between these phenomena are pointed out.

## 1 Introduction

Colloidal nanocrystals of lead mixed-halide perovskites (LMHPs), such as the cesium-based ternary inorganic compounds CsPbX_3_ (X = mixed Cl/Br and Br/I systems), have garnered significant attention for their wide-ranging applications.^1^ These materials are particularly promising for use in solar cells,^2,3^ light-emitting diodes (LEDs),^4−7^ and high-efficiency photodetectors,^8,9^ owing to their exceptional fluorescence quantum efficiency and ionic charge carrier conduction.^10−12^ Their optoelectronic versatility, driven by the tunable absorption and emission across the ultraviolet–visible (UV–vis) spectrum via halide composition adjustment in the PbX_3_ octahedral framework, further enhances their potential in various technological domains.^13−16^

Recently, LMHP materials have emerged as a promising alternative to highly crystalline silicon currently used in solar panel production. These materials have quickly increased in efficiency from 3.8% to 25.6%,^17,18^ sparking a technological race to commercialize them. Despite their high efficiency in converting solar energy to electricity, perovskites face long-term performance issues because of the halide migration processes. Achieving long-term operational stability and enhancing the conversion efficiency of LMHP-based photovoltaic devices,^19−22^ and light-emitting diodes remain a significant challenge. This is primarily due to the intrinsic softness of their crystal lattice and carrier recombination.^21,22^

The main questions regarding the halide migration dynamics in perovskite bulk crystals or nanocrystals involve light-induced phase segregation (PS) and reconstruction (PR) phenomena. PS is typically associated with the formation of Br-rich and I-rich domains,^23−25^ characterized by a red shift of the photoluminescence (PL) spectrum, restoring the original mixed halide composition (blue shift) when kept in the dark.^26^ The PL red shift occurs because there is fast transport and recombination of charge carriers toward the I-rich regions due to the reduction in the bandgap.^27,28^ Recently, many models have been proposed to describe the PS process. The most accepted models are related to ion migration due to defect-driven^29^ and polaron-induced lattice strain-driven halide segregation in bulk perovskites.^28^ In both mechanisms, the light-induced PS occurs at grain boundaries rather than grains. On the other hand, light-induced phase segregation in nanocrystals seems to be associated with dimensionality effects. For example, G. Reyes et al.^30^ proposed controlling the light-induced phase segregation in CsPbBr_3-x_I_*x*_ thin films through nanocrystal size engineering. They observed that phase segregation occurs above a threshold size of 46(7) nm, and developed a model based on the charge carrier diffusion length to explain light-induced halide segregation. In contrast, M. L. Crawford et al.^31^ observed the PS phenomenon in cubic MAPb(I_*x*_Br_1–*x*_)_3_ nanocrystals (NCs) with an average edge length of approximately 16 nm. According to this study, light-induced iodine migration occurs from the surface to the core of particles during PS.^31^ Moreover, PS is mainly reported in bulk perovskites, and its underlying mechanisms are slightly different from those observed in nanocrystals.^32,33^

More recently, Zhe Li et al.^29^ pioneeringly demonstrated the phase reconstruction phenomenon in MAPb(I_1–*x*_Br_*x*_)_3_ thin films (single crystals), in which the expulsion of iodide ions occurs shortly after the onset of the phase segregation, leading to a complete MAPbBr_3_ phase reconstruction. Such a process proved to be irreversible. On the other hand, the selective expulsion of iodide has been previously reported in mixed halide perovskite films in contact with a solvent.^34,35^ However, the kinetics of ion migration in thin films of colloidal mixed-halide perovskite NCs during phase segregation and reconstruction is not yet wholly understood. Notwithstanding, photoinduced halide segregation and phase reconstruction have a potentially negative impact on photovoltaic performance, leading to ion accumulation on electrode surfaces, polarization effects, and halide degradation.^24^ At the same time, these light-induced effects represent an excellent opportunity to understand fundamental aspects such as defect-induced halide migration,^36−38^ polaron generation,^39,40^ exciton diffusion in low-dimensional materials,^41,42^ and superfluorescence.^43−45^ In this context, we combined hyperspectral fluorescence microspectroscopy and computational methods to elucidate the underlying mechanism of phase reconstruction and segregation in CsPbBr_3-x_I_*x*_ (0 < *x* < 3) NC thin films.

## 2 Results and Discussion

### 2.1 Optical Properties of CsPbBr_3-x_I_*x*_ Nanocrystal Thin Films

**Figure 1:**
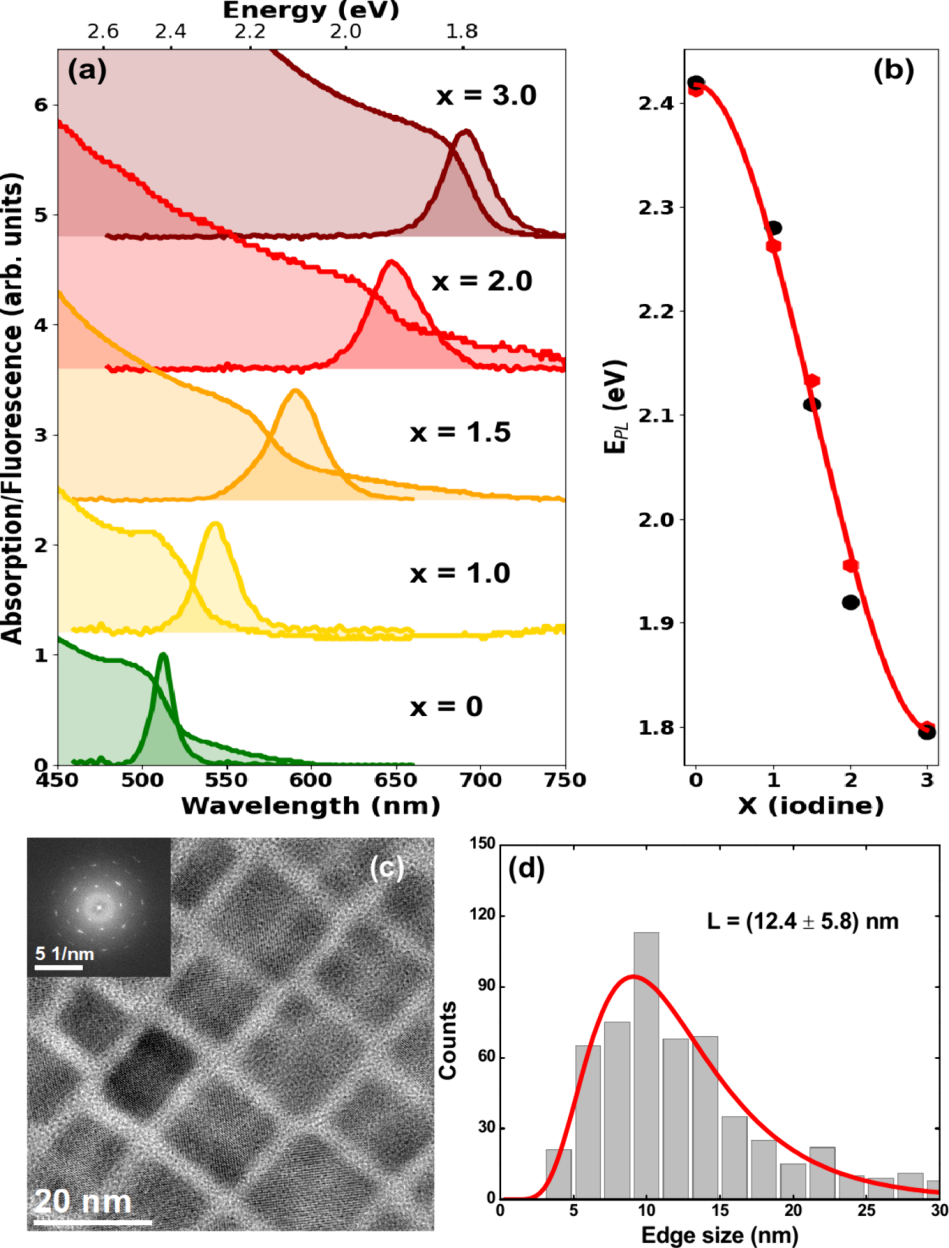
(a) Absorption and emission spectra for CsPbBr_3-x_I_*x*_ NC thin films with different compositions (x = 0, 1.0, 1.5, 2.0, and 3.0). (b) PL peak energy as a function of the iodine fraction for the LMHP NCs obtained here (black circles) and from the data reported by G. Nedelcu et al.^46^ (red diamonds). (c) Illustrative TEM image for the synthesized CsPbBrI_2_ NCs and (d) corresponding TEM particle size distribution histogram fitted to a log-normal function (red curve) with average edge length L = (12.4 ± 5.8) nm.

Figure 1(a) shows the ground-state absorption and fluorescence spectra of CsPbBr_3-x_I_*x*_ NC thin films with different compositions: x = 0 (CsPbBr_3_), x = 1 (CsPbBr_2_I), x = 1.5 (CsPbBr_1.5_I_1.5_), x = 2 (CsPbBrI_2_), and x = 3 (CsPbI_3_). The NCs were ligated with oleylamine and oleic acid molecules in order to improve the chemical stability and reduce surface defects. The first excitonic transition extracted from the minimum of the second order derivative of the absorption data is located at 539 nm (2.30 eV) for CsPbBr_2_I, 571 nm (2.17 eV) for CsPbBr_1.5_I_1.5_, and 622 nm (2.00 eV) for CsPbBrI_2_ thin films, whereas for the pure Br (x = 0) and I (x = 3) compositions, we have 504 nm (2.46 eV) and 690 nm (1.84 eV), respectively. For the fluorescence spectra compiled in Figure 1(a), the CsPbBr_3-x_I_*x*_ NC thin films present a narrow emission line width in the visible region (40–80 meV). Figure 1(b) (red diamonds) illustrates the relationship between the PL peak energy and the iodine fraction in solution-grown LMHP NCs obtained from the data reported by G. Nedelcu et al.^46^ Thus, we estimated the halide fraction in each sample from the photon energy corresponding to the PL peak position (E_PL_) according to the following analytical equation: E_PL_ (eV) = 2.42 – 0.2x^2^ + 0.04x^3^, which was used to fit the experimental data of ref (46) (red line).

As can be seen, our fluorescence peak position outcomes (black circles) are in agreement with these fitting data, with the emission peaks close to 513 nm (2.42 eV), 543 nm (2.28 eV), 590 nm (2.10 eV), 646 nm (1.92 eV), and 690 (1.80 eV), for x = 0, 1.0, 1.5, 2.0, and 3.0, respectively. It is worth mentioning that the emission peak values measured for the pure Br and I compositions will be used as input parameters for the linear spectral unmixing computational method, as described in Section 4.5. Figure 1(c) shows an illustrative transmission electron microscope (TEM) image of the synthesized CsPbBrI_2_ nanocrystals, highlighting their cubic structure, while Figure 1(d) presents the corresponding particle size distribution histogram. The TEM images and the X-ray diffractogram (XRD) patterns indicate that the average edge length of the cube-shaped nanocrystals is between 7 and 14 nm, depending on the composition. As reported in many papers,^1,15,47−49^ the NC size tends to increase as a function of the iodine content. The TEM images, XRD patterns and the time-resolved fluorescence curves can be seen in Figures S1–S2 and Table S1 of the Supporting Information (SI).

### 2.2 Irreversible Phase Reconstruction in CsPbBr_2_I and CsPbBr_1.5_I_1.5_ Nanocrystal Films

**Figure 2:**
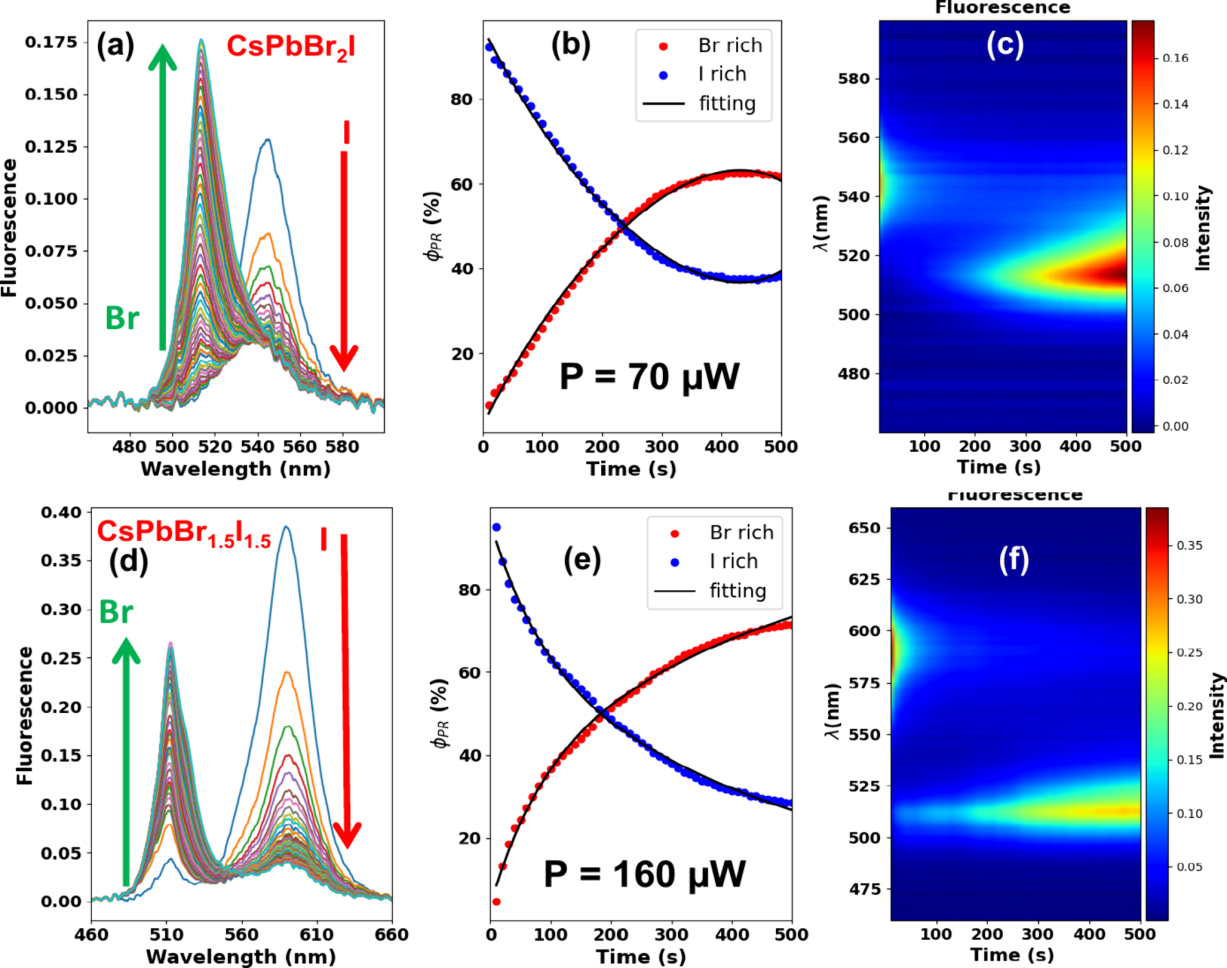
Fluorescence spectra over excitation time for (a) CsPbBr_2_I and (d) CsPbBr_1.5_I_1.5_ nanocrystal thin films with different compositions. (b, e) PR (Phase Reconstruction) kinetic curves for the Br-rich (red circles) and I-rich (blue circles) pure phase emissions obtained by the linear unmixing method applied to the fluorescence curves in (a) and (d), respectively. (c, f) Fluorescence colormaps emphasize the PR process.

Figure 2 illustrates the halide kinetics for the (a) CsPbBr_2_I_1_ and (d) CsPbBr_1.5_I_1.5_ nanocrystal thin films using hyperspectral fluorescence microscopy. We stopped the measurements at 500 s because photobleaching occurs after the fluorescence saturates at 510 nm, depending on the laser irradiance. As can be seen, immediately after the laser excitation, the fluorescence signal related to the mixed-halide perovskite at 545 nm (Figure 2(a)) and 585 nm (Figure 2(d)) decreased and another band raised at ∼510 nm for both samples. The laser power P used to monitor the fluorescence kinetics of the irradiated perovskite thin films is indicated in Figure 2(a-c) (CsPbBr_2_I) and Figure 2(d-f) (CsPbBr_1.5_I_1.5_). The observed temporal evolution of the fluorescence spectrum under laser irradiation is related to the CsPbBr_3_ nanocrystal phase recovery. In this sense, the laser promotes the expulsion of I^–^ from the perovskite lattice and generates a reconstruction of Br-rich domains, forming the CsPbBr_3_ phase. As previously mentioned, such a process is known as phase reconstruction. Figures 2(b) and 2 (e) depict the relative increase and reduction in the pure emissions of these Br-rich or I-rich phases obtained from the linear spectral unmixing method, respectively. We defined a figure of merit, φ_PR_, from the linear spectral unmixing analysis related to the phase reconstruction yield after 500 s of laser excitation. To obtain the average PR rates, the relative increase and decrease curves were modeled using a biexponential function (k*_Br_* = [1 – (*k*_01_e^(±*k*_1_t)^ + *k*_02_e^(±*k*_2_t)^)] and k*_I_* = *k*_01_e^(±*k*_1_t)^ + *k*_02_e^(±*k*_2_t)^, solid lines in Figure 2(b) and (e)), which is characterized by fast and slow rate constants, represented by *k*_1_ and *k*_2_, respectively, with *k*_01_ and *k*_02_ as pre-exponential factors. These values can be found in the Table S2 of the Supporting Information. Finally, Figures 2(c) and (f) provide fluorescence colormaps for the described PL behavior, highlighting the increase in the bromine phase compared to the iodine phase due to laser excitation. In contrast to what Z. Li et.al.^29^ observed, phase segregation did not occur in our experiments with the CsPbBr_2_I and CsPbBr_1.5_I_1.5_ nanocrystalline films with mixed halide compositions. Such behavior can possibly be attributed to spatial dimension effects because we are studying thin films composed of perovskite nanocrystals, that is, nanometer-sized particles with finite physical dimensions, rather than bulk perovskite crystals.

**Figure 3:**
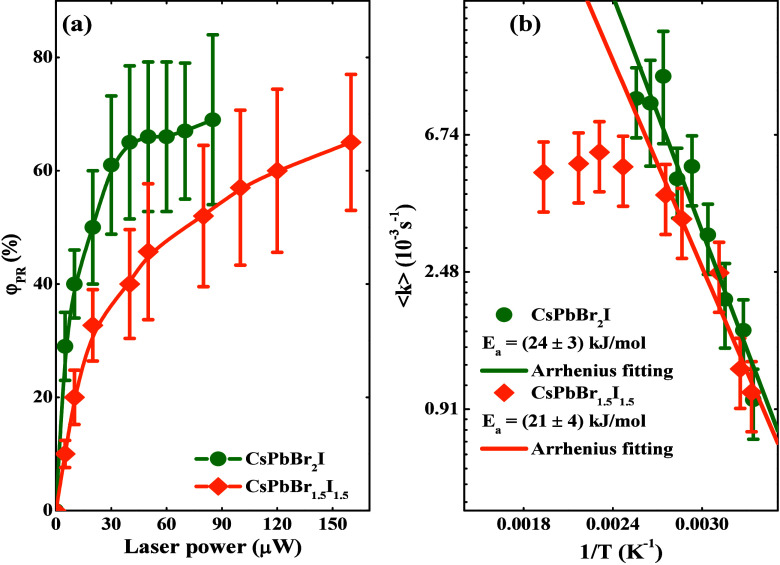
Phase reconstruction yield (a) for the CsPbBr_2_I (green circles) and CsPbBr_1.5_I_1.5_ (orange rhombuses) thin film samples and (b) their respective Arrhenius plot (log–linear plot) and fitting curve (straight lines).

To shed more light on these outcomes and contribute to elucidating the phase reconstruction activation pathways in our perovskite films with different Br/I halide compositions, we conducted experiments by changing the laser power from 10 μW up to 80 μW for CsPbBr_2_I, and from 10 μW up to 160 μW for CsPbBr_1.5_I_1.5_ (corresponding to irradiances from 6.37 × 10^2^ W/cm^2^ to 1.02 × 10^4^ W/cm^2^, considering the laser waist radius estimated at 1 μm using the zero-damage method^50^). Thus, the laser-induced final temperature was determined by simulating heat propagation in the irradiated thin film, employing the Fourier Law and the finite-difference method (see SI for details).^51^Figure 3 (a) illustrates the PR yield (φ_PR_) as a function of the laser power. As clearly noticed, the PR yield rises by increasing the laser power and the Br halide concentration in the fabricated film. However, the PR effect saturates for sufficiently high laser powers at ∼70% for both nanocrystal thin film compositions. In fact, in films with higher Br content, the amount of iodine required to be removed from the nanocrystal lattice via laser irradiation is smaller, thereby facilitating the process. In this context, we calculate the activation energy (*E*_a_) from the Arrenhius Law, i.e., *k* = *k*_0_*e*^–*E*_a_/*k*_B_*T*^, in which the average PR rate constant ⟨k_PR_⟩ values were taken from the relationship ⟨k_PR_⟩ = (*k*_01_*k*_1_+*k*_02_*k*_2_)/(*k*_01_+*k*_02_), *T* is the laser-induced temperature, *k*_B_ is the Boltzmann constant, and *k*_*0*_ is a pre-exponential factor. Figure 3 (b) illustrates the Arrhenius plot for the CsPbBr_2_I and CsPbBr_1.5_I_1.5_ thin films, which provides *E*_a_ = (24 ± 3) kJ/mol (∼248 ± 30 meV) and (21 ± 4) kJ/mol (∼217 ± 42 meV) for these respective compositions. Therefore, the estimated *E*_*a*_ values did not differ significantly between the samples with different halide compositions. These results agree with the vacancy-mediated hopping barriers in mixed halide perovskites.^23^

**Figure 4:**
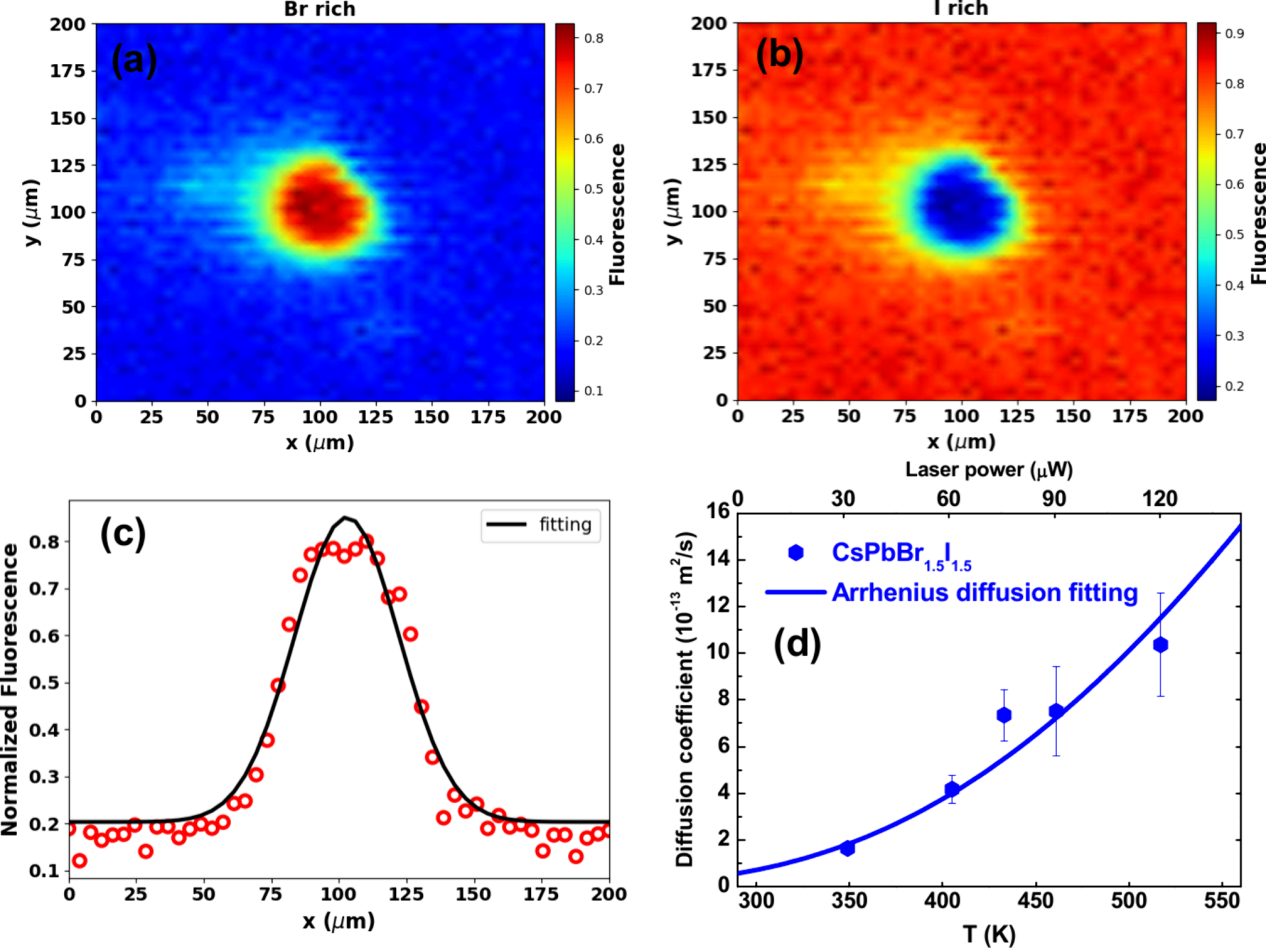
Hyperspectral fluorescence mapping at a 200 × 200 μm area around the laser photoexcited region obtained from the linear unmixing method for (a) Br-rich and (b) I-rich regions in the CsPbBr_1.5_I_1.5_ thin film using a 120 μW laser power. (c) Fluorescence intensity profile as an *x*-axis translation length function (red circles, y coordinate fixed at 100 μm) and its respective fitting curve employing eq 1 (solid line). (d) Experimental D_PR_ values (PR diffusion coefficient) as a function of the laser-induced temperature. The solid line represents the fitting curve obtained using the Arrhenius equation for diffusibility.

Because the mixed halide dynamics in perovskites have a strong transient character, hyperspectral fluorescence microspectroscopy was applied to probe the reversible nature of the PR process and determine its microscale length. For this purpose, we scanned a 200 μm × 200 μm area around the laser incident region after 500 s of exposure. Figure 4 presents the colormaps acquired by hyperspectral fluorescence microspectroscopy for the CsPbBr_1.5_I_1.5_ thin film using a 120 μW laser power. Concerning the hyperspectral fluorescence images, it was possible to apply the linear spectral unmixing method to decompose the emission of each halide phase separately. Figure 4 shows the colormap for the (a) Br-rich and (b) I-rich regions obtained for the CsPbBr_1.5_I_1.5_ film sample using a laser power of 120 μW. In these colormaps, the blue areas in Figure 4 (a) and the red areas in Figure 4(b) represent regions where no changes occurred in the CsPbBr_1.5_I_1.5_ nanocrystal thin film emission. Similar colormaps were obtained for the CsPbBr_2_I film sample. As can be seen, around the laser excitation region (center of the map), a halo of inhibition of iodine (Figure 4(b)) and rich in bromine (Figure 4(a)) is formed. This interesting outcome shows that the PR process has an irreversible character, indicating that iodine is removed from the nanocrystal structure and degraded when interacting with the environment and high-intensity laser due to its instability in the thin film.^29^

Another interesting aspect to highlight in Figure 4(a) is that the Br-rich halo is much larger than the laser beam diameter (∼2 μm), indicating that PR phenomenon has photo and thermal activation. Moreover, we have observed that the halo diameter increases as a function of the laser power. Because the PR involves halide ion diffusion, we consider Fick’s second law of diffusion to determine the PR diffusion coefficient (D_PR_, (m^2^/s)) using the following relationship:
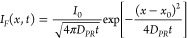
1in which *I*_*F*_(*x*, *t*) is the fluorescence signal along space and time, *t* = 1/⟨*k*⟩is the characteristic time for the photothermal effect obtained from Figure 2(b,e), or *t* = τ is the fluorescence lifetime (see Figure S3 and Table S3 of the Supporting Information) for the purely electronic effect, and *I*_0_ is a fitting parameter related to the fluorescence amplitude. Figure 4(c) reports the fluorescence intensity profile as an *x*-axis translation length function (red circles, y coordinate fixed at 100 μm) and its respective fitting curve obtained by employing eq 1 (solid line). In Figure 4(d), we present the PR diffusion coefficient (D_PR_) values (diamonds) found from eq 1 fitting analysis as a function of the laser-induced temperature. The solid line illustrates the fitting curve corresponding to the Arrhenius equation for diffusibility, i.e., D_PR_ = D_0_e^(−Ea/k*_B_*T)^, in which D_0_ = 5.3 × 10^–11^ m^2^/s is the retrivied pre-exponential factor and *E*_a_ is the retrieved activation energy, which is in agreement with the results obtained from Figure 3 (*E*_a_= (182 ± 21) meV, while for Figure 3 we found *E*_a_ = (217 ± 42) meV). Therefore, the Arrhenius model for ion diffusibility describes our experimental results well within the standard deviation of the different optical measurements. In this case, using this model, we can recover the D_PR_ value at room temperature (293 K), i.e., D_PR_ (293 K) = 6.3 × 10^–14^ m^2^/s, which is of the order of the Br–I interdiffusion coefficient in quasi-2D perovskite thin films,^52^ corroborating our interpretation of the PR process.

**Figure 5:**
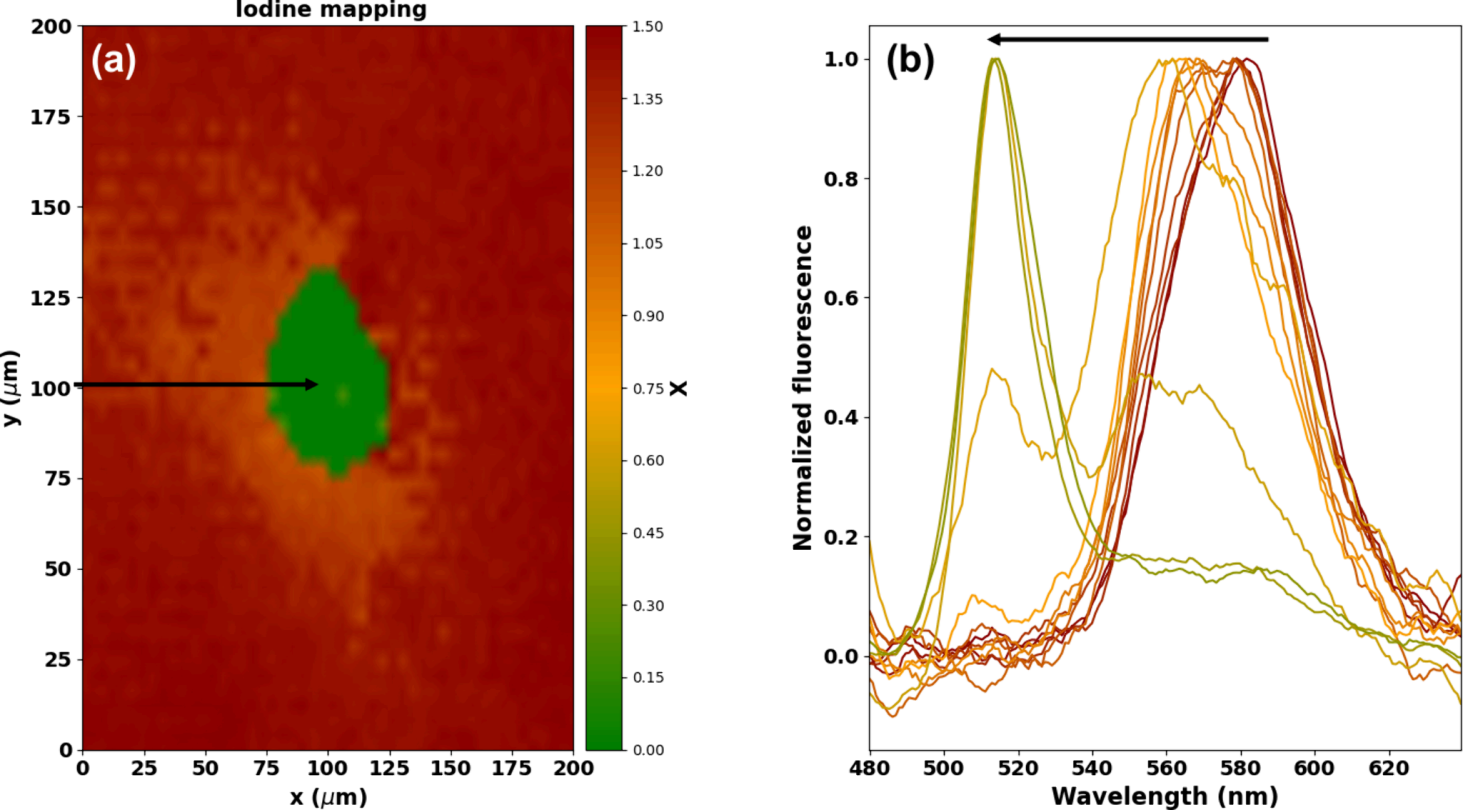
(a) Iodine fraction colormap for the CsPbBr_1.5_I_1.5_ thin film obtained from the analytical equation for the composition-dependent fluorescence peak position. (b) The arrows show the fluorescence spectra for each position of the colormap along the x-translation axis (y coordinate set at 100 μm).

Hyperspectral fluorescence microscopy allows us to explore the halide diffusion in the fabricated perovskite films. For example, in Figure 5 (a), we show the colormap for the CsPbBr_1.5_I_1.5_ thin film resolved in iodine fraction (color bar represents the iodine fraction value between x = 0 and x = 1.5) for the 200 μm × 200 μm area around the laser excitation site. To that end, we employed the analytical equation for the composition-dependent fluorescence peak position E_PL_(x) (see Figure 1(b)) to retrieve the iodine fraction. The excitation (located at the central region of the colormap) presents a green halo with iodine content close to zero, indicating the CsPbBr_3_ phase reconstruction. Figure 5(b) illustrates the fluorescence spectra for each *x*-axis translation length with the y coordinate fixed at 100 μm. As noted, we clearly see that the wavelength at which the fluorescence is maximum (peak wavelength) is blue-shifted from the borders to the center. These results indicate that the PR process occurs with less efficiency radially from the laser excitation region due to the lower photothermal effect. Therefore, increasing the bromine concentration in the nanocrystal structure changes the fluorescence peak position, which undergoes a continuous blue shift toward the 510 nm emission wavelength assigned to the CsPbBr_3_ reconstructed phase, thereby forwarding an intermediary photoconversion state for the neighboring nanocrystals in the thin film. These outcomes corroborate the interplay between photo and thermal activation during the PR process.

In summary, we proposed a model to explain the phase reconstruction observed in cesium lead mixed-halide perovskite nanocrystal thin films. Upon laser excitation, light induces charge carrier diffusion within the nanocrystal core and surface in a process that occurs as rapidly as the exciton lifetime. As reported by Zhe Li,^29^ charge carriers interact with iodide ions (I^–^) and tend to oxidize them, forming iodine(I) and/or triiodide ions (I_3_^–^). This process can occur both in the bulk and on the surface. However, in small nanocrystals with high surface-to-volume ratios, continuous excitation causes iodine to migrate to the surface, which becomes unstable in thin films, leading to degradation and the formation of iodide vacancies due to laser irradiance. All these processes are light-driven and, therefore, strongly localized at the laser spot, given that the charge carrier diffusion lengths are tens or hundreds of nanometers.^30,41,53^ However, as shown in this work, the phase reconstruction extends beyond the laser spot area (Figures 4 and 5), suggesting that temperature plays an important role. Meanwhile, the laser-induced temperature promotes a substantial migration from the Br^–^ ion toward the I^–^ vacancy, following an Arrhenius-like diffusion behavior, as shown in Figure 4 (d). After several hundred seconds, the green emission associated with the CsPbBr_3_ phase dominates the nanocrystal emission at x = 1.0 and 1.5 compositions, with a halo extending to the micrometer scale. Notably, phase segregation was not observed in our nanocrystal samples, which contrasts with the work of Z. Li et al.^29^ for a perovskite single crystal. This is probably because our nanocrystal samples have a small edge length, which means a high surface-to-volume ratio, favoring the expulsion of iodine even before seeing the iodine local enrichment.

**Figure 6:**
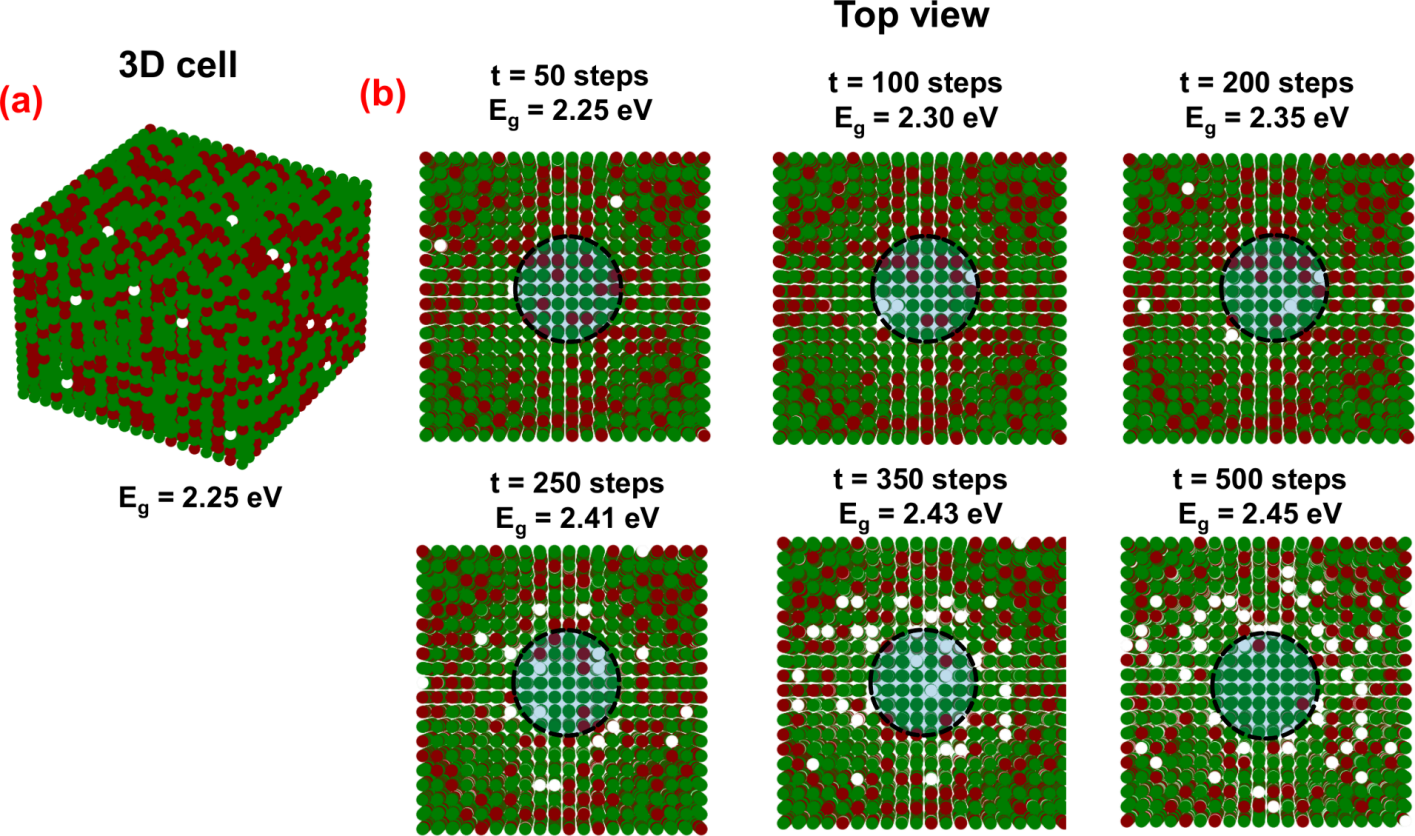
(a). Kinetic Monte Carlo (KMC) simulations employing a 3D cell containing a 20 × 20 × 20 (N_*x*_ x N_*y*_ x N_*z*_) grid, in which circles in green, red, and white colors represent bromide, iodide and vacancies, respectively. (b) The top view of the 3D cell illustrates the different time steps used in the KMC simulations.

To gain a deeper understanding of this PR phenomenon, we employed the Kinetic Monte Carlo (KMC) method to simulate the phase reconstruction process based on these results. Figure 6(a) showcases the 3D cell containing a 20 × 20 × 20 (N_*x*_ × N_*y*_ × N_*z*_) grid, in which the green, red, and white colored circles indicate the bromide, iodide ions and vacancies, respectively. Before the irradiation, the grid presents a stochastic distribution of iodide (35.2%) and bromide (64%) ions and vacancies (0.8%) to reproduce the pristine films.^54^ A Gaussian beam laser was simulated with a waist radius of 0.1N around the center of the grid, as shown by the dashed circle in the center of each simulation cell in Figure 6(b). The migration rate (*k*_m_) or local hopping barrier associated with halide migration from an occupied to a vacant site was determined using the Arrhenius equation with the activation energy obtained in the present work. The KMC model is detailed in Section 4.6. Figure 6(b) shows the top view of the 3D cell. Under laser excitation, the inward and outward diffusion of bromide and iodide ions induce iodide inhibition and bromide-rich halo formation over time due to laser irradiation. Consequently, the bandgap energy of the excitation region changes from 2.27 to 2.45 eV, reproducing our experimental results shown in Figures 4 and 5 where the CsPbBr_3_ pure phase reconstruction is quantified. In fact, the bandgap of the irradiated subcell computed at the longest simulation time (E_g_ = 2.45 eV, t = 500 steps) agrees quantitatively with the CsPbBr_3_ nanocrystals bandgap (L∼ 10 nm). Also, according to our KMC simulations, this local phase reconstruction effect does not occur if the hopping barrier for iodide is higher than that for bromide, corroborating our results.

### 2.3 Reversible Phase Segregation in CsPbBrI_2_ Thin Films

**Figure 7:**
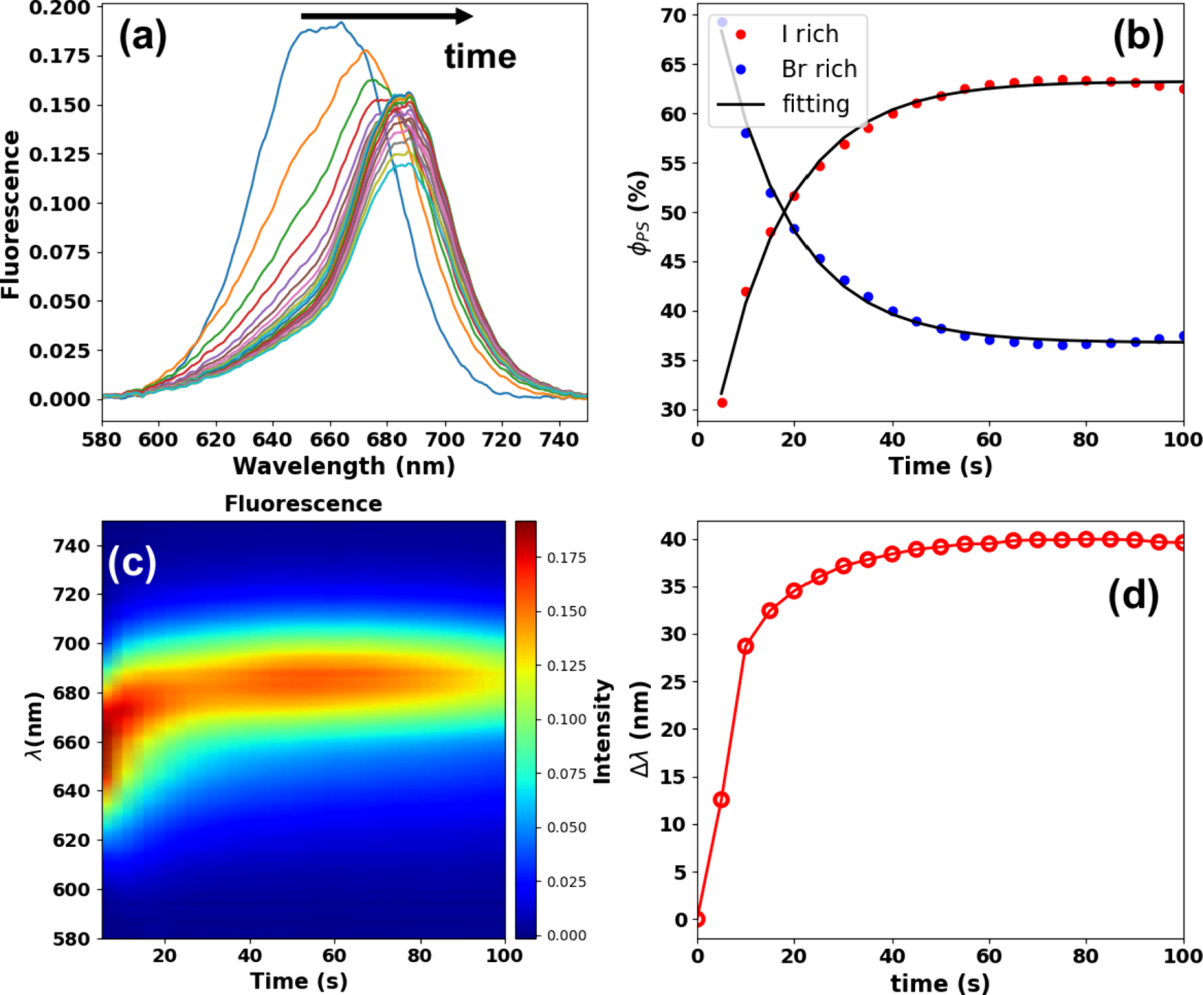
(a) Fluorescence spectra over excitation time for the CsPbBrI_2_ nanocrystal thin film. (b) Phase segregation (PS) kinetic curves were retrieved using the linear unmixing method applied to the fluorescence spectra in (a). (c) Fluorescence colormap emphasizing the PS process. (d) Wavelength change (fluorescence peak position variation) over excitation time during the PS process (extracted from the spectroscopic data in (a)).

We have performed the same experiments reported in Figure 2 for the CsPbBrI_2_ thin films. However, we did not observe any effect related to phase reconstruction for this sample. On the other hand, the phase segregation takes place with high efficiency. Figure 7(a) shows the fluorescence spectra over the excitation time for CsPbBrI_2_ thin films prepared, using the cw-laser power of 300 μW for 100 s at 405 nm (excitation wavelength). As noted, the fluorescence exhibited a red shift from 650 to 688 nm, approaching the pure CsPbI_3_ emission at ∼ 690 nm, which characterizes the light-induced halide segregation. Moreover, the fluorescence intensity decreases due to the lower fluorescence quantum yield of the CsPbI_3_ compared to the CsPbBrI_2_ nanocrystals. Such an effect was not observed in our CsPbBr_2_I and CsPbBr_1.5_I_1.5_ nanocrystal samples when performing the same experiment. Therefore, these outcomes indicate that NC size and iodine composition are essential physicochemical parameters to be considered in our analysis. In fact, CsPbBrI_2_ nanocrystals were larger than those deposited in the thin film samples in which we observed phase reconstruction (at least two times larger in volume; see SI). Also, the higher iodine content in our CsPbBrI_2_ thin film sample decreases the bandgap, thus favoring the phase segregation induced by defect- or polaron-driven processes.^40,55,56^ However, the average edge length of these nanocrystals is still substantially smaller than the threshold size of 46(7) nm established by G. Reyes et al.^30^ A possible explanation for this effect is the significant difference between the laser intensities used in our work and those reported in ref.^30^ For example, we employed laser intensities at least a thousand times higher.

To interpret these results, we calculated the steady-state carrier density achieved under Gaussian cw-laser excitation using the following rate equation model:^28^
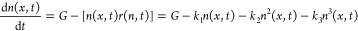
2with
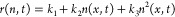
3
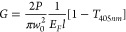
4in which *r*(*n*, *t*) is the total recombination rate, *G* is the exciton generation rate (exciton/cm^3^.s), *P* (50 μW) is the average laser power, *w*_0_ stands for the Gaussian laser waist (1 × 10^–4^ cm),  is the film thickness (∼40 × 10^–7^ cm), *E*_*F*_ is the excitation photon energy (in Joule), *T*_405 nm_ is the thin film transmittance at 405 nm, *n*(*x*, *t*)is the charge carrier density. In addition, *k*_1_ is the exciton recombination rate obtained from the fluorescence decay time τ_fluo_ (*k*_1_∼1/τ_fluo_), *k*_2_ is the bimolecular charge recombination rate constant, and *k*_3_ is the Auger recombination rate constant. eq 2 was numerically solved using the explicit finite difference method in 1D. We could not find the parameters *k*_2_ and *k*_3_ for the CsPbBrI_2_ thin films, so we used the rate constant values for high-quality CsPbBr_3_ (10 nm) nanocrystal thin films reported in ref.^57^ (*k*_2_ = 5.0 x10^–11^ cm^3^s^–1^; *k*_3_ = 0.16 x10^–28^ cm^6^ s^–1^). Based on this model, we found a steady-state carrier density of 1.7 × 10^18^ cm^–3^ within a 20 ns temporal window. Thus, we can estimate the charge carrier diffusion length (*L*_*D*_) as
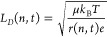
5in which μ is the effective sum mobilities of excited electrons and holes, *T* is the temperature, *k*_B_ is the Boltzmann constant, and *e* is the elementary charge. We have used μ = 3 cm^2^/V·s obtained from photoconductivity measurements with excitation at 400 nm. This value comes from ref.,^57^ which is the same one employed to obtain the parameters *k*_2_ and *k*_3_. Following this approach, we found *L*_*D*_ = 10.2 nm, which is very close to the edge length of our CsPbBrI_2_ nanocrystals (L_CsPbBrI2_ = (12.4 ± 5.8) nm). In this case, the charge carriers remain localized within the nanocrystal, reinforcing the observed phase segregation. In fact, phase segregation introduces potential barriers and traps for charge carriers, shortening their diffusion length. Therefore, larger particle sizes, combined with iodine content and high laser intensities, can modify the transport and recombination by reducing the diffusion length of charge carriers and bandgap.^41,42,53,58^ As a matter of fact, the NC size is not the only factor responsible for the transition between phase reconstruction and segregation.

In Figure 7(b), we again employed the linear unmixing method to obtain the light-induced phase segregation yield, φ_PS_. As can be seen, the CsPbBrI_2_ phase decreases at the expense of the CsPbI_3_ phase formation. The fluorescence colormap for the PS behavior highlighting the iodine enrichment is depicted in Figure 7(c) and (d), which show the wavelength change Δλ (magnitude of the fluorescence peak position red shift) during the PS process obtained from the Gaussian decomposition method, in which the CsPbBrI_2_ emission initially localized at ∼650 nm approaches the pure CsPbI_3_ emission at ∼690 nm (Δλ∼40 nm) in a short excitation time (50 s).

**Figure 8:**
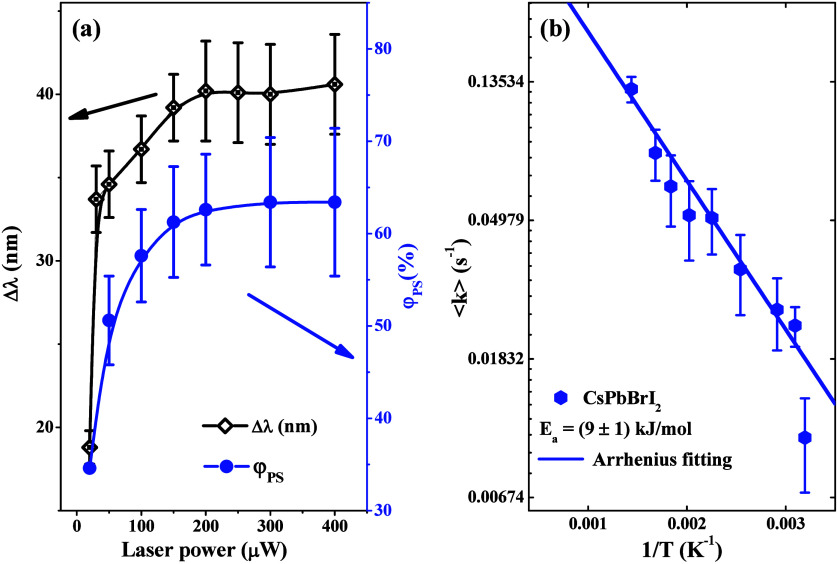
(a) Phase segregation yield (φ_PS_, blue circles) and red shift of the emission peak position (Δλ, open rhombuses) as functions of the laser power, and (b) the Arrhenius plot (blue hexagons) for the CsPbBrI_2_ thin film (average phase segregation rate constant ⟨k⟩ vs laser-induced reciprocal temperature (1/T)).

Mao, W. X. et al.^28^ reported that phase segregation in perovskite single crystals could be a light-driven reversal phenomenon. According to them,^28^ the PS process at high carrier densities (high light intensities) decreases the carrier-induced strain gradients, causing an overlap of the polarons responsible for the reversal phase segregation. In this context, we performed PS experiments for several cw-laser powers, from 20 μW to 400 μW, as shown in Figure 8(a). It is possible to observe that the phase segregation yield (blue circles) associated with iodine enrichment increases monotonically up to around 70%. The same behavior was observed for the emission wavelength red shift (black rhombuses) as a function of the laser excitation power. Therefore, in our outcomes, the light-induced reversal PS is not triggered. The fabrication process for nanocrystal thin films by spin-coating most probably introduces steric hindrance, leading to the formation of trap states that weaken polaron overlap and, consequently, hinder the reversal halide segregation process. This issue is especially significant in nanocrystals because their higher surface-to-volume ratio results in a much greater density of trap states than single crystals.^59,60^ In Figure 8(b), we presented the Arrhenius plot and estimated the activation energy of (9 ± 1) kJ/mol ((93 ± 10) meV) for phase segregation in the CsPbBrI_2_ nanocrystalline thin film. This value is smaller than those obtained for PS in perovskite single crystals.^23^ This is probably due to the high laser irradiance used in our experiments, which contributes to the occurrence of a high-efficiency PS process and reduces the charge carrier diffusion length, as previously shown.

**Figure 9:**
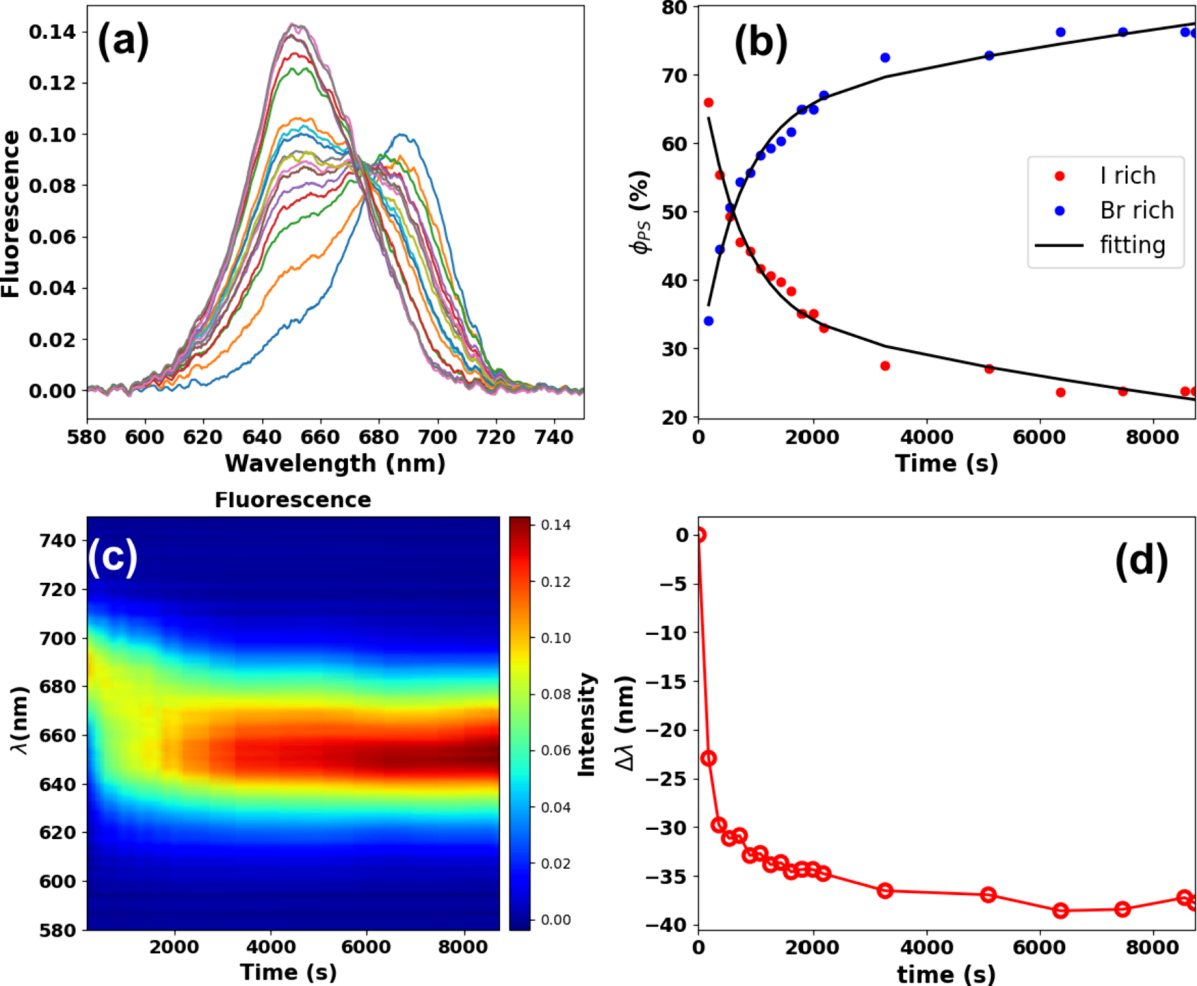
(a) Dark recovery of the PL spectra over time, (b) phase segregation (PS) kinetics for the Br-rich and I-rich phase emissions obtained from the linear unmixing analysis, (c) color mapping emphasizing the PS process, (d). Blue shift over time for the dark recovery process in the CsPbBrI_2_ nanocrystal thin film.

Figure 9 presents our results from fluorescence microspectroscopy experiments conducted on a CsPbBrI_2_ film to confirm the reversible nature of phase segregation in iodine-rich nanocrystal thin films. To observe the recovery of the phase segregation after laser irradiation, the fluorescence spectra were recorded for approximately 2.5 h in dark ambient conditions, with measurements taken every 5 min using low laser power and an integration time of 1 s. Figure 9(a) demonstrates the fluorescence spectra recovery with the PL peak position blue-shifting from 688 to 650 nm. From the linear unmixing method, we determined the kinetic curves for the return of the CsPbBrI_2_ phase, as shown in Figure 9(b). PS dark recovery is emphasized by the fluorescence colormap in Figure 9(c), resulting in an approximate Δλ = −38 nm (emission wavelength blue shift at the final observation time *t* ∼ 9000 s), as illustrated in Figure 9(d). Therefore, our results show a complete dark recovery of the PS phenomenon in nanocrystal thin films, similar to that observed in perovskite single crystals.^28^

Based on these results, we proposed a model to explain the phase segregation phenomenon in mixed-halide perovskite nanocrystals under high-intensity laser exposure. As mentioned, the CsPbBrI_2_ nanocrystals are larger than the CsPbBr_2_I and CsPbBr_1.5_I_1.5_ nanocrystals (at least two times higher in volume). In this case, the higher volume available increases the probability of the iodine migration from the surface to the core^31^ because, as shown, the diffusion of charge carriers occurs within the nanocrystal due to their small diffusion length (L_D_ = 10.2 nm). At the same time, the iodide-rich domain presents the valence band maximum position (VBM = −5.44 eV) and conduction band minimum (CBM = −3.45 eV) with energies higher than the band edges of bromide-rich regions (VBM = −6.00 and CBM = −3.60),^61^ which favors the formation of “type-II heterostructure”, with an iodide-rich phase within a bromide-rich lattice.^31^ In this configuration, the hole is confined in the iodide-phase valence band (“core”) while the electron is in the bromide-phase conduction band (“shell”). As seen, increasing the iodide content causes a more pronounced red shift in the VBM than the CBM, explaining the phase segregation phenomena. In this case, the CsPbBrI_2_ “type-II heterostructure” allows the bromide ions to shield the iodide ions from environmental factors and laser-induced degradation. Consequently, the irreversible bromide phase formation due to iodide photodegradation (phase reconstruction) does not occur. In addition, the PS process is reversible, which means that the probability of the iodide ion migrating to the NC surface is low because it becomes very unstable due to its laser and environment interaction, which would culminate in its photodegradation. Concomitantly, upon high intensity excitation, the laser-induced temperature promotes the formation of perovskite quantum dot solids due to ligand degradation (oleylamine and oleic acid), making them closer to each other. Quantum dot solids^62^ are formed by reducing the interdot spacing, which enhances the optical interactions between the nanocrystals. The formation of perovskite quantum dot solids decreases the surface area-to-volume ratio at the short-range length in the thin film. Concomitantly, the quantum dot solid formation may also promote the development of grain boundaries, which are regions susceptible to phase separation in perovskite single crystals.^28^ These processes collectively promote phase segregation rather than phase reconstruction via defect- or polaron-driven mechanisms.

As mentioned, photoinduced halide segregation and phase reconstruction can have a potentially negative impact on photovoltaic device performance. Based on our findings, several strategies can be employed to mitigate these undesirable effects. For example, adjusting the halide ratio can help balance lattice strain, thereby reducing halide segregation and phase reconstruction effects.^63^ Additionally, as reported by many researchers,^64,65^ applying a protective polymer coating to perovskite thin films can shield them from environmental factors, enhancing the performance and durability of optoelectronic devices such as solar cells, lasers, and LEDs. Furthermore, using small nanocrystals with a narrow size distribution, combined with low irradiance, can increase the charge-carrier diffusion length, stabilize the thin film bandgap over a long-range length, and consequently improve device stability.

## 3 Conclusion

Herein, we used hyperspectral fluorescence microspectroscopy and computational modeling to elucidate the complex ion migration dynamics in mixed-halide CsPbBr_3-x_I_*x*_ (0 < *x* < 3) perovskite nanocrystal thin films. Specifically, for x = 1.0 and x = 1.5, we observed halide migration leading to local bromide enrichment and photoluminescence blue shifts from 545 to 510 nm due to irreversible phase reconstruction, forming CsPbBr_3_ domains. Photothermal effects drive this phenomenon. By applying linear spectral unmixing and kinetic analyses, we determined crucial thermodynamic parameters such as halide activation energy and diffusion coefficient, correlating these findings with Fick’s second law. Moreover, we have shown that the phase reconstruction diffusion presents an Arrhenius-like behavior as a function of the laser-induced temperature. Thus, we retrieved the room temperature Br–I interdiffusion coefficient of 6.2 × 10^–14^ m^2^/s, consistent with quasi-2D perovskite films. In contrast, for x = 2.0, larger CsPbBrI_2_ nanocrystals exhibited phase segregation rather than reconstruction, with iodide ions migrating from the surface to the core due to NC size, iodine content, and high laser power that combined leads to a small charge-carrier diffusion length, culminating in high PS yield. We also display real-time dark recovery for light-induced halide phase segregation. On the other hand, the light-induced reversal halide segregation was not observed in our samples, probably because of the less efficient process of overlapping the polarons in perovskite nanocrystals than in the single crystals. Our outcomes provided deeper insights into the halide migration mechanisms and transitions between these phenomena. This study enhances the understanding of ion kinetics in mixed-halide perovskites and paves the way for optimizing their optoelectronic properties for advanced applications.

## 4 Materials and Methods

### 4.1 Chemical Synthesis of Colloidal Perovskite Nanocrystals

The following precursors and reagents were used for synthesizing perovskite nanocrystals: cesium carbonate (Cs_2_CO_3_, Sigma-Aldrich, 99.9%), lead(II) bromide (PbBr_2_, Sigma-Aldrich, 99%), lead(II) iodide (PbI_2_, Sigma-Aldrich, 99%), oleic acid (OA, Sigma-Aldrich, 90%), 1-octadecene (ODE, Sigma-Aldrich, 90%), oleylamine (OAm, Sigma-Aldrich, 90%), isopropyl alcohol (Dinâmica, 99.5%), and toluene (Synth, 99.5%).

A previously described method was used to produce colloidal nanocrystals of cesium lead mixed-halide perovskites.^1^ Initially, a cesium oleate solution was prepared. For this purpose, 0.25 mmol of Cs_2_CO_3_, 4 mL of ODE, and 0.25 mL of OA were added to a 3-neck flask. This mixture was degassed and dried under vacuum at 120 °C for 60 min. The system was then heated under argon flow at 150 °C until all Cs_2_CO_3_ had completely reacted with OA. This precursor solution can be stored for subsequent syntheses, taking care to heat it to 100 °C before use. To prepare CsPbBr_3-x_I_*x*_ NCs (0 < *x* < 3), PbBr_2_ and PbI_2_ at different concentrations were used, varying the value of x (x = 0, 1, 1.5, 2, 3). When x = 0 (CsPbBr_3_), 0.188 mmol of PbBr_2_ was used. In addition to the salts, 5.0 mL ODE, 0.5 mL OA and 0.5 mL OAm were added to the 3-neck flask. This mixture was dried under vacuum at 120 °C for 1 h, then heated to 150 °C under argon gas. Finally, 0.4 mL of the previously prepared Cs oleate solution was injected under an argon atmosphere and, after 10 s, the solution was cooled in an ice bath for 5 s. For the purification step, the suspension was transferred to a Falcon tube and mixed with 7.5 mL of isopropyl alcohol, followed by centrifugation at 9000 rpm for 30 min. The obtained NCs were suspended in toluene.

### 4.2 Production of Perovskite Films

Perovskite thin films were fabricated via spin coating on microscope slides, as per our previous work.^51^ First, the slides were subjected to a sequence of 10 min ultrasonic baths with the following reagents: ultrapure water, ethanol, isopropanol, and acetone. They underwent a 10 min treatment with a plasma cleaner to finish cleaning the slides. After air drying, previously prepared nanocrystal suspension (see Section 4.1 for synthesis details) was applied to the substrate using a spin coating in two steps: first, 400 rpm for 30 s, and then 6000 rpm for 10 s. The resulting thin film was stored in an inert atmosphere in a glovebox for later analysis.

### 4.3 Hyperspectral Fluorescence Microscopy and Optical Characterization

The thin film analyses were initially performed on an Agilent Cary 5000 UV–vis-NIR spectrophotometer with a diffuse reflectance accessory (DRA) to confirm the semiconductor properties of NC thin film samples. Hyperspectral fluorescence microscopy experiments were carried out using a continuous-wave (cw) laser emitting light at 405 nm (excitation wavelength) with TEM_00_ mode, a microscope objective with 40× magnification, a numerical aperture of 0.65, and a working distance of 0.6 mm. The details of this experimental setup can be found in SI. Hyperspectral images were captured using low laser power and a fast scanning laser (200 ms acquisition per spectrum) to mitigate issues with fluorescence scattering and prevent any sample alteration over time.

Time-resolved photoluminescence measurements were conducted on thin film samples using a Horiba Fluorolog-3 Jobin Yvon spectrofluorimeter. Excitation was achieved using a 455 nm pulsed nanoLED, and all decay curves were recorded at room temperature.

### 4.4 TEM and XRD Measurements

The samples were characterized using a JEM 21 00 FEG - TEM microscope operating at 200 kV. The samples after being dropped (3 μL) onto the 400 match carbon grid covered with ultrathin carbon film (∼3 nm), the samples were placed in the Plasma Cleaner Model 1020 – Instruments Fischione for 10 to 20 s and then the analyses were carried out. The measurements were performed at LNNano, CNPEM in Campinas - SP. The observed particles were counted using the ImageJ software.

The crystalline structure of the perovskite nanocrystal thin films was characterized by XRD. The diffraction patterns were obtained on a Shimadzu XRD - 6000 diffractometer, with Cu K α radiation, λ = 1.5418 Å, generated at 30 kV and with a current of 30 mA, 1° min^–1^ scan speed, in the range of 2θ = 10° – 70°. The Inorganic Crystal Structure Database (ICSD) n° 14610 confirmed the formation of cubic structures (as can be seen in SI).

### 4.5 Linear Spectral Unmixing Method

Linear spectral unmixing is a technique used to break down the total detected fluorescence signal (*S*) into contributions from different light-emitting chromophores. In our study, we used this method to distinguish between the emissions from Br-rich (*F*_*Br*_) and I-rich (*F*_*I*_) phases in mixed halide perovskite thin films.^66−68^ Thus:
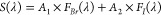
6in which *A*_*1*_ and *A*_*2*_ are the amplitudes of the specific contributions of bromide and iodide ions, respectively, and *F*_*Br*_ and *F*_*I*_ are the reference fluorescence spectra of the CsPbBr_1–*x*_I_*x*_ nanocrystalline thin films, shown in Figure 1. In this case, to quantify the contribution of each phase separately, *A*_*1*_ and *A*_*2*_, the method employed a least-squares approach to minimize the squared differences between calculated and measured values, based on the following set of differential equations:
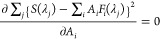
7

Here *j* represents the number of wavelengths considered and *i* represents the number of emissive species. Therefore, we solved eq 7 with the singular value decomposition method employed by the Python software. Consequently, we found the contribution (%) of the Br-rich and I-rich phases during the phase reconstruction and segregation kinetic experiments.

### 4.6 Kinetic Monte Carlo Simulations

We used the 3D Kinetic Monte Carlo (KMC) method^69,70^ to model the phase reconstruction phenomenon. The simulation was conducted on a 20 × 20 × 20 site grid, where each site randomly allocated one atom of Br, I, or a halide vacancy. Reflecting experimental conditions in an air-saturable atmosphere, we set the Br, I, and vacancy concentrations at 64%, 35.2%, and 0.8%, respectively. The migration or hop rate (*k*_*m*_) for Br and I ions was determined using the Arrhenius equation:
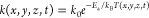
8in which *k*_0_is the pre-exponential factor, *E*_*a*_ is the activation energy or the local hopping barrier associated with halide migration from an occupied to a vacant site (*E*_*Br*_ = 0.21 eV and *E*_*I*_ = 0.10 eV were obtained in this work), *k*_*B*_ is the Boltzmann constant, and *T* is the laser-induced temperature. The laser-induced temperature was calculated using the following equation:
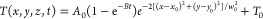
9in which *A*_*0*_ is the maximum laser-induced temperature amplitude calculated from the Fourier Law using the finite difference method (see SI). *B* is the heating rate and *T*_*0*_ is the room temperature. *w*_0_ stands for the Gaussian laser waist radius and *x*_0_ and *y*_0_ indicate the grid positions where the laser is incident.

For each instant of time t, the local bandgap E_g_(t) of a region surrounding the center of the simulation cube (0.2N_*x*_ × 0.2N_*y*_ × 0.2N_*z*_), which has been irradiated within the volume V_irradiated_ = 0.2N_*x*_ × 0.2N_*y*_ × N_*z*_, is calculated based on the halide composition through the following equation obtained in this work: E_g_(t) = 2.46 – 1.78x^2^ + 1.16x^3^, where x is the relative concentration of the iodide ion, i.e., x = (C_I_+C_V_)/(C_Br_+C_I_+C_V_), and C represents the concentration of the halides (I, Br) and vacancies (V). The bandgap calculation takes into consideration the vacancy concentration because, in our case, we are interested in the bandgap modification due to the enrichment of bromide ions. Therefore, the simple substitution of an iodine atom by a vacancy does not alter the bandgap. The volume of all subcells is equal to the volume of the total cell. Thus, the vacancy percentage in the 3D cell is given by n_v_(%) = [1 – (V_subcell_/V_cell_)].

The vacancy position matrix is initialized at each step of the KMC simulation. The algorithm searches for ions in the first neighbors of each vacancy.The neighbor to which the vacancy will hop was chosen based on the migration rate for the neighbor normalized by the total migration rate for each vacancy. The probability P of a vacancy-neighbor swap was determined by comparing the ratio of the Arrhenius rates in the subcell around the vacancy (V_subcell_ = 0.2N_*x*_ × 0.2N_*y*_ × 0.2N_*z*_) and the Arrhenius rates in total volume (V_cell_ = N_*x*_ × N_*y*_ × N_*z*_), i.e., . The swap happens if the probability exceeds a drawn random number. In this case, the hopping probability indirectly considers the local bandgap effect. Additionally, during the phase reconstruction event, the iodine atom can escape, leaving behind a vacancy. This process is driven by the laser-induced temperature and is calculated based on the following equation:
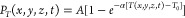
10in which α and *A* are experimental parameters. Then, a random number is drawn and compared to the probability threshold (*P*_*T*_) to determine whether the neighboring iodine atom converts to a halide vacancy, thereby updating the vacancy position.

In order to obtain the distribution of halide ions and vacancies at a particular time in the grid, the number of hops per unit time for each vacancy is given by the inverse of the sum of the Arrhenius rates for all neighbors within the subcell volume (). Each time step corresponds to *Δt* = 1. The bromide, iodide, and vacancy concentrations were monitored in the laser excitation region, and the grid stability was established when the excitation region bandgap reached a *plateau*.

## Data Availability

The data that support the findings of this study are available from the corresponding author upon reasonable request.

## Supporting Information Available

TEM images, XRD patterns, and time-resolved fluorescence curves for the CsPbBr_3-x_I_*x*_ spin-coated nanocrystalline thin films. A computational model based on the finite-difference method was employed to estimate the laser-induced temperature. (PDF)

## References

[ref1] Protesescu L.; Yakunin S.; Bodnarchuk M. I.; Krieg F.; Caputo R.; Hendon C. H.; Yang R. X.; Walsh A.; Kovalenko M. V. Nanocrystals of Cesium Lead Halide Perovskites (CsPbX3, X = Cl, Br, and I): Novel Optoelectronic Materials Showing Bright Emission with Wide Color Gamut. Nano Lett. 2015, 15 (6), 3692–3696. 10.1021/nl5048779. 25633588 PMC4462997

[ref2] Kojima A.; Teshima K.; Shirai Y.; Miyasaka T. Organometal Halide Perovskites as Visible-Light Sensitizers for Photovoltaic Cells. J. Am. Chem. Soc. 2009, 131 (17), 6050 10.1021/ja809598r. 19366264

[ref3] Noh J. H.; Im S. H.; Heo J. H.; Mandal T. N.; Seok S. I. Chemical Management for Colorful, Efficient, and Stable Inorganic-Organic Hybrid Nanostructured Solar Cells. Nano Lett. 2013, 13 (4), 1764–1769. 10.1021/nl400349b. 23517331

[ref4] Gao L.; Zhang Y. L.; Gou L. J.; Wang Q.; Wang M.; Zheng W. T.; Wang Y. H.; Yip H. L.; Zhang J. Q. High efficiency pure blue perovskite quantum dot light-emitting diodes based on formamidinium manipulating carrier dynamics and electron state filling. Light-Science & Applications 2022, 11 ( (1), ). 10.1038/s41377-022-00992-5. PMC9747997 36513629

[ref5] Hu J. C.; Bi C. H.; Ren K.; Zhang X. T.; Wang W. Q.; Ma S.; Wei M. Z.; Lu Y.; Sui M. L. High-Efficiency Pure-Red CsPbI _3_ Quantum Dot Light-Emitting Diodes Enabled by Strongly Electrostatic Potential Solvent and Sequential Ligand Post-treatment Process. Nano Lett. 2024, 24 (15), 4571–4579. 10.1021/acs.nanolett.4c00651. 38565076

[ref6] Li X. M.; Wu Y.; Zhang S. L.; Cai B.; Gu Y.; Song J. Z.; Zeng H. B. CsPbX _3_ Quantum Dots for Lighting and Displays: Room-Temperature Synthesis, Photoluminescence Superiorities, Underlying Origins and White Light-Emitting Diodes. Adv. Funct. Mater. 2016, 26 (15), 2435–2445. 10.1002/adfm.201600109.

[ref7] Wan Q.; Huang L.; Kong L.; Zhang Q. G.; Zhang C. Y.; Liu M. M.; Liao X. R.; Zhan W. J.; Zheng W. L.; Yuan C. W.; et al. High-Efficiency Semitransparent Light-Emitting Diodes with Perovskite Nanocrystals. ACS Appl. Mater. Interfaces 2022, 14 (17), 19697–19703. 10.1021/acsami.2c01665. 35438955

[ref8] Wang H.; Kim D. H. Perovskite-based photodetectors: materials and devices. Chem. Soc. Rev. 2017, 46 (17), 5204–5236. 10.1039/C6CS00896H. 28795697

[ref9] Xie C.; Liu C. K.; Loi H. L.; Yan F. Perovskite-Based Phototransistors and Hybrid Photodetectors. Adv. Funct. Mater. 2020, 30 (20), 1903907 10.1002/adfm.201903907.

[ref10] Zhang Q.; Yin Y. D. All-Inorganic Metal Halide Perovskite Nanocrystals: Opportunities and Challenges. Acs Central Science 2018, 4 (6), 668–679. 10.1021/acscentsci.8b00201. 29974062 PMC6026778

[ref11] Wu P.; Gangadharan D. T.; Saidaminov M. I.; Tan H. R. A Roadmap for Efficient and Stable All-Perovskite Tandem Solar Cells from a Chemistry Perspective. Acs Central Science 2023, 9 (1), 14–26. 10.1021/acscentsci.2c01077. 36712494 PMC9881206

[ref12] Ferreira D. L.; Silva A. G.; Schiavon M. A.; Vivas M. G. Determination of the particle size distribution of cube-shaped colloidal perovskite quantum dots from photoluminescence spectra: A combined theoretical-experimental approach. J. Chem. Phys. 2024, 161 ( (16), ). 10.1063/5.0234432. 39450726

[ref13] Akkerman Q. A.; D’Innocenzo V.; Accornero S.; Scarpellini A.; Petrozza A.; Prato M.; Manna L. Tuning the Optical Properties of Cesium Lead Halide Perovskite Nanocrystals by Anion Exchange Reactions. J. Am. Chem. Soc. 2015, 137 (32), 10276–10281. 10.1021/jacs.5b05602. 26214734 PMC4543997

[ref14] Bartel C. J.; Sutton C.; Goldsmith B. R.; Ouyang R. H.; Musgrave C. B.; Ghiringhelli L. M.; Scheffler M. New tolerance factor to predict the stability of perovskite oxides and halides. Science Advances 2019, 5 ( (2), ). 10.1126/sciadv.aav0693. PMC6368436 30783625

[ref15] Diroll B. T.; Nedelcu G.; Kovalenko M. V.; Schaller R. D. High-Temperature Photoluminescence of CsPbX3 (X = Cl, Br, I) Nanocrystals. Adv. Funct. Mater. 2017, 27 (21), 1606750 10.1002/adfm.201606750.

[ref16] Liao M. L.; Shan B. B.; Li M. In Situ Raman Spectroscopic Studies of Thermal Stability of All-Inorganic Cesium Lead Halide (CsPbX _3_ , X = Cl, Br, I) Perovskite Nanocrystals. J. Phys. Chem. Lett. 2019, 10 (6), 1217–1225. 10.1021/acs.jpclett.9b00344. 30821150

[ref17] Laboratory N. R. E. Best Research-Cell Efficiencies. 2024. https://www.nrel.gov/pv/cell-efficiency.html (accessed.

[ref18] Saliba M.; Matsui T.; Seo J. Y.; Domanski K.; Correa-Baena J. P.; Nazeeruddin M. K.; Zakeeruddin S. M.; Tress W.; Abate A.; Hagfeldt A.; et al. Cesium-containing triple cation perovskite solar cells: improved stability, reproducibility and high efficiency. Energy Environ. Sci. 2016, 9 (6), 1989–1997. 10.1039/C5EE03874J. 27478500 PMC4936376

[ref19] Carvalho T. A. D.; Magalhaes L. F.; Santos C. I. D.; de Freitas T. A. Z.; Vale B. R. C.; da Fonseca A. F. V.; Schiavon M. A. Lead-Free Metal Halide Perovskite Nanocrystals: From Fundamentals to Applications. Chem.—Eur. J. 2023, 29 ( (4), ). 10.1002/chem.202202518. 36206198

[ref20] Li X.; Yang J. Y.; Jiang Q. H.; Chu W. J.; Zhang D.; Zhou Z. W.; Ren Y. Y.; Xin J. W. Enhanced photovoltaic performance and stability in mixed-cation perovskite solar cells via compositional modulation. Electrochim. Acta 2017, 247, 460–467. 10.1016/j.electacta.2017.07.040.

[ref21] Singh S.; Moons E. Impact of photoinduced phase segregation in mixed-halide perovskite absorbers on their material and device stability. APL Energy 2024, 2, 10.1063/5.0190465.

[ref22] Safdari M.; Kim D.; Balvanz A.; Kanatzidis M. G. Mitigation of Halide Segregation by Cation Composition Management in Wide Bandgap Perovskites. ACS Energy Letters 2024, 9, 3400–3408. 10.1021/acsenergylett.4c01281.

[ref23] Brennan M. C.; Draguta S.; Kamat P. V.; Kuno M. Light-Induced Anion Phase Segregation in Mixed Halide Perovskites. Acs Energy Letters 2018, 3 (1), 204–213. 10.1021/acsenergylett.7b01151.

[ref24] Cho J. S.; Kamat P. V. Photoinduced Phase Segregation in Mixed Halide Perovskites: Thermodynamic and Kinetic Aspects of Cl-Br Segregation. Advanced Optical Materials 2021, 9 (18), 2001440 10.1002/adom.202001440.

[ref25] Gautam S. K.; Kim M.; Miquita D. R.; Bouree J. E.; Geffroy B.; Plantevin O. Reversible Photoinduced Phase Segregation and Origin of Long Carrier Lifetime in Mixed-Halide Perovskite Films. Adv. Funct. Mater. 2020, 30 (28), 2002622 10.1002/adfm.202002622.

[ref26] Athapaththu D. V.; Kordesch M. E.; Chen J. X. Monitoring Phase Separation and Dark Recovery in Mixed Halide Perovskite Clusters and Single Crystals Using *In Situ* Spectromicroscopy. J. Phys. Chem. Lett. 2024, 15 (4), 1105–1111. 10.1021/acs.jpclett.3c03280. 38262449 PMC10877542

[ref27] Yoon S. J.; Draguta S.; Manser J. S.; Sharia O.; Schneider W. F.; Kuno M.; Kamat P. V. Tracking Iodide and Bromide Ion Segregation in Mixed Halide Lead Perovskites during Photoirradiation. Acs Energy Letters 2016, 1 (1), 290–296. 10.1021/acsenergylett.6b00158.

[ref28] Mao W. X.; Hall C. R.; Bernardi S.; Cheng Y. B.; Widmer-Cooper A.; Smith T. A.; Bach U. Light-induced reversal of ion segregation in mixed-halide perovskites. Nat. Mater. 2021, 20 (1), 55–61. 10.1038/s41563-020-00826-y. 33077949

[ref29] Li Z.; Zheng X.; Xiao X.; An Y. K.; Wang Y. B.; Huang Q. Y.; Li X.; Cheacharoen R.; An Q. Y.; Rong Y. G.; . Beyond the Phase Segregation: Probing the Irreversible Phase Reconstruction of Mixed-Halide Perovskites. Advanced Science 2022, 9 ( (5), ). 10.1002/advs.202103948. PMC8844510 34923773

[ref30] Gualdrón-Reyes A. F.; Yoon S. J.; Barea E. M.; Agouram S.; Muñoz-Sanjosé V.; Meléndez A. M.; Niño-Gómez M. E.; Mora-Seró I. Controlling the Phase Segregation in Mixed Halide Perovskites through Nanocrystal Size. Acs Energy Letters 2019, 4 (1), 54–62. 10.1021/acsenergylett.8b02207. 30662954 PMC6333216

[ref31] Crawford M. L.; Sadighian J. C.; Hassan Y.; Sadhanala A.; Nawab L.; Wong C. Y. Formation of Iodide-Rich Domains During Halide Segregation in Lead-Halide Perovskite Nanocrystals. J. Phys. Chem. Lett. 2023, 14, 8962–8969. 10.1021/acs.jpclett.3c02068. 37772502

[ref32] Motti S. G.; Krieg F.; Ramadan A. J.; Patel J. B.; Snaith H. J.; Kovalenko M. V.; Johnston M. B.; Herz L. M. CsPbBr _3_ Nanocrystal Films: Deviations from Bulk Vibrational and Optoelectronic Properties. Adv. Funct. Mater. 2020, 30 (19), 1909904 10.1002/adfm.201909904.

[ref33] Motti S. G.; Patel J. B.; Oliver R. D. J.; Snaith H. J.; Johnston M. B.; Herz L. M. Phase segregation in mixed-halide perovskites affects charge-carrier dynamics while preserving mobility. Nat. Commun. 2021, 12 ( (1), ). DOI: 10.1038/s41467-021-26930-4. PMC8630172 34845219

[ref34] Cho J. S.; DuBose J. T.; Mathew P. S.; Kamat P. V. Electrochemically induced iodine migration in mixed halide perovskites: suppression through chloride insertion. Chem. Commun. 2021, 57 (2), 235–238. 10.1039/D0CC06217K. 33305300

[ref35] DuBose J. T.; Mathew P. S.; Cho J. S.; Kuno M.; Kamat P. V. Modulation of Photoinduced Iodine Expulsion in Mixed Halide Perovskites with Electrochemical Bias. J. Phys. Chem. Lett. 2021, 12 (10), 2615–2621. 10.1021/acs.jpclett.1c00367. 33689371

[ref36] Knight A. J.; Wright A. D.; Patel J. B.; McMeekin D. P.; Snaith H. J.; Johnston M. B.; Herz L. M. Electronic Traps and Phase Segregation in Lead Mixed-Halide Perovskite. Acs Energy Letters 2019, 4 (1), 75–84. 10.1021/acsenergylett.8b02002.

[ref37] Wang W. T.; Chiang C. H.; Zhang Q.; Mu Y. J.; Wu C. G.; Feng S. P. Defect-Induced Dipole Moment Change of Passivators for Improving the Performance of Perovskite Photovoltaics. Acs Energy Letters 2024, 9 (6), 2982–2989. 10.1021/acsenergylett.4c00839.

[ref38] Wright A. D.; Patel J. B.; Johnston M. B.; Herz L. M. Temperature-Dependent Reversal of Phase Segregation in Mixed-Halide Perovskites. Adv. Mater. 2023, 35 (19), 2210834 10.1002/adma.202210834. 36821796

[ref39] Bischak C. G.; Wong A. B.; Lin E.; Limmer D. T.; Yang P. D.; Ginsberg N. S. Tunable Polaron Distortions Control the Extent of Halide Demixing in Lead Halide Perovskites. J. Phys. Chem. Lett. 2018, 9 (14), 3998–4005. 10.1021/acs.jpclett.8b01512. 29979045

[ref40] Zhang H.; Park N. G. Polarons in perovskite solar cells: effects on photovoltaic performance and stability. Journal of Physics-Energy 2023, 5 (2), 024002 10.1088/2515-7655/acb96d.

[ref41] Seitz M.; Magdaleno A. J.; Alcázar-Cano N.; Meléndez M.; Lubbers T. J.; Walraven S. W.; Pakdel S.; Prada E.; Delgado-Buscalioni R.; Prins F. Exciton diffusion in two-dimensional metal-halide perovskites. Nat. Commun. 2020, 11 ( (1), ). 10.1038/s41467-020-15882-w. PMC7184754 32341361

[ref42] Shcherbakov-Wu W.; Saris S.; Sheehan T. J.; Wong N. N.; Powers E. R.; Krieg F.; Kovalenko M. V.; Willard A. P.; Tisdale W. A. Persistent enhancement of exciton diffusivity in CsPbBr _3_ nanocrystal solids. Science Advances 2024, 10 ( (8), ). 10.1126/sciadv.adj2630. PMC10881049 38381813

[ref43] Rainò G.; Becker M. A.; Bodnarchuk M. I.; Mahrt R. F.; Kovalenko M. V.; Stöferle T. Superfluorescence from lead halide perovskite quantum dot superlattices. Nature 2018, 563 (7733), 671 10.1038/s41586-018-0683-0. 30405237

[ref44] Tang Y.; Jing Y.; Sum T. C.; Bruno A.; Mhaisalkar S. G. Superfluorescence in Metal Halide Perovskites. Adv. Energy Mater. 2025, 15, 2400322 10.1002/aenm.202400322.

[ref45] Krieg F.; Sercel P. C.; Burian M.; Andrusiv H.; Bodnarchuk M. I.; Stöferle T.; Mahrt R. F.; Naumenko D.; Amenitsch H.; Rainò G.; et al. Monodisperse Long-Chain Sulfobetaine-Capped CsPbBr _3_ Nanocrystals and Their Superfluorescent Assemblies. Acs Central Science 2021, 7 (1), 135–144. 10.1021/acscentsci.0c01153. 33532576 PMC7845019

[ref46] Nedelcu G.; Protesescu L.; Yakunin S.; Bodnarchuk M. I.; Grotevent M. J.; Kovalenko M. V. Fast Anion-Exchange in Highly Luminescent Nanocrystals of Cesium Lead Halide Perovskites (CsPbX3, X = Cl, Br, I). Nano Lett. 2015, 15 (8), 5635–5640. 10.1021/acs.nanolett.5b02404. 26207728 PMC4538456

[ref47] Akkerman Q. A.; Nguyen T. P. T.; Boehme S. C.; Montanarella F.; Dirin D. N.; Wechsler P.; Beiglböck F.; Rainò G.; Erni R.; Katan C.; et al. Controlling the nucleation and growth kinetics of lead halide perovskite quantum dots. Science 2022, 377 (6613), 1406–1412. 10.1126/science.abq3616. 36074820

[ref48] Diroll B. T.; Nedelcu G.; Kovalenko M. V.; Schaller R. D. High-Temperature Photoluminescence of CsPbX _3_ (X = Cl, Br, I) Nanocrystals.Adv. Funct. Mater. 2017, 27 ( (21), ). 10.1002/adfm.201606750.

[ref49] Nedelcu G.; Protesescu L.; Yakunin S.; Bodnarchuk M. I.; Grotevent M. J.; Kovalenko M. V. Fast Anion-Exchange in Highly Luminescent Nanocrystals of Cesium Lead Halide Perovskites (CsPbX _3_ , X = Cl, Br, I). Nano Lett. 2015, 15 (8), 5635–5640. 10.1021/acs.nanolett.5b02404. 26207728 PMC4538456

[ref50] Liu J. M. Simple Technique for Measurements of Pulsed Gaussian-Beam Spot Sizes. Opt. Lett. 1982, 7 (5), 196–198. 10.1364/OL.7.000196. 19710869

[ref51] de Souza G. F. d.; Magalhães L. F.; de Souza Carvalho T. A. d.; Ferreira D. L.; Pereira R. S.; da Cunha T. R.; Bettini J.; Schiavon M. A.; Vivas M. G. Probing the cw-Laser-Induced Fluorescence Enhancement in CsPbBr3 Nanocrystal Thin Films: An Interplay between Photo and Thermal Activation. ACS Appl. Mater. Interfaces 2024, 16, 34303 10.1021/acsami.4c03934. 38885089 PMC11231974

[ref52] Akriti; Zhang S.; Lin Z.-Y.; Shi E.; Finkenauer B. P.; Gao Y.; Pistone A. J.; Ma K.; Savoie B. M.; Dou L. Quantifying Anionic Diffusion in 2D Halide Perovskite Lateral Heterostructures. Adv. Mater. 2021, 33 (51), 2105183 10.1002/adma.202105183.

[ref53] Xiao X.; Wu M.; Ni Z. Y.; Xu S.; Chen S. S.; Hu J.; Rudd P. N.; You W.; Huang J. S. Ultrafast Exciton Transport with a Long Diffusion Length in Layered Perovskites with Organic Cation Functionalization. Adv. Mater. 2020, 32 (46), 2004080 10.1002/adma.202004080. 33048430

[ref54] Ruth A.; Brennan M. C.; Draguta S.; Morozov Y. V.; Zhukovskyi M.; Janko B.; Zapol P.; Kuno M. Vacancy-Mediated Anion Photosegregation Kinetics in Mixed Halide Hybrid Perovskites: Coupled Kinetic Monte Carlo and Optical Measurements. Acs Energy Letters 2018, 3 (10), 2321–2328. 10.1021/acsenergylett.8b01369.

[ref55] Mosconi E.; Meggiolaro D.; Snaith H. J.; Stranks S. D.; De Angelis F. Light-induced annihilation of Frenkel defects in organo-lead halide perovskites. Energy Environ. Sci. 2016, 9 (10), 3180–3187. 10.1039/C6EE01504B.

[ref56] Pols M.; Brouwers V.; Calero S.; Tao S. X. How fast do defects migrate in halide perovskites: insights from on-the-fly machine-learned force fields. Chem. Commun. 2023, 59 (31), 4660–4663. 10.1039/D3CC00953J. 36994486

[ref57] Motti S. G.; Krieg F.; Ramadan A. J.; Patel J. B.; Snaith H. J.; Kovalenko M. V.; Johnston M. B.; Herz L. M. CsPbBr _3_ Nanocrystal Films: Deviations from Bulk Vibrational and Optoelectronic Properties. Adv. Funct. Mater. 2020, 30 ( (19), ). 10.1002/adfm.201909904.

[ref58] Dong Q. F.; Fang Y. J.; Shao Y. C.; Mulligan P.; Qiu J.; Cao L.; Huang J. S. Electron-hole diffusion lengths > 175 mu m in solution-grown CH3NH3PbI3 single crystals. Science 2015, 347 (6225), 967–970. 10.1126/science.aaa5760. 25636799

[ref59] Zheng X. P.; Hou Y.; Sun H. T.; Mohammed O. F.; Sargent E. H.; Bakr O. M. Reducing Defects in Halide Perovskite Nanocrystals for Light-Emitting Applications. J. Phys. Chem. Lett. 2019, 10 (10), 2629–2640. 10.1021/acs.jpclett.9b00689. 31038960

[ref60] Swartwout R.; Hoerantner M. T.; Bulovic V. Scalable Deposition Methods for Large-Area Production of Perovskite Thin Films. Energy & Environmental Materials 2019, 2 (2), 119–145. 10.1002/eem2.12043.

[ref61] Aldakov D.; Reiss P. Safer-by-Design Fluorescent Nanocrystals: Metal Halide Perovskites vs Semiconductor Quantum Dots. J. Phys. Chem. C 2019, 123 (20), 12527–12541. 10.1021/acs.jpcc.8b12228.

[ref62] Kagan C. R.; Murray C. B. Charge transport in strongly coupled quantum dot solids. Nat. Nanotechnol. 2015, 10 (12), 1013–1026. 10.1038/nnano.2015.247. 26551016

[ref63] Mallem K.; Prodanov M. F.; Dezhang C.; Marus M.; Kang C. B.; Shivarudraiah S. B.; Vashchenko V. V.; Halpert J. E.; Srivastava A. K. Solution-Processed Red, Green, and Blue Quantum Rod Light-Emitting Diodes. ACS Appl. Mater. Interfaces 2022, 14 (16), 18723–18735. 10.1021/acsami.2c04466. 35417119

[ref64] Wang Z.; He H. Y.; Liu S.; Wang H.; Zeng Q. S.; Liu Z.; Xiong Q. H.; Fan H. J. Air Stable Organic-Inorganic Perovskite Nanocrystals@Polymer Nanofibers and Waveguide Lasing. Small 2020, 16 ( (43), ). 10.1002/smll.202004409. 33006251

[ref65] Fallah K.; Alam S. N.; Ghaffary B.; Yekekar A.; Aghiyan S.; Taravati S. Enhancement of the environmental stability of perovskite thin films via AZ5214-photoresist and PMMA coatings. Optical Materials Express 2024, 14 (8), 2083–2094. 10.1364/OME.532998.

[ref66] Dickinson M. E.; Bearman G.; Tille S.; Lansford R.; Fraser S. E. Multi-spectral imaging and linear unmixing add a whole new dimension to laser scanning fluorescence microscopy. Biotechniques 2001, 31 (6), 1272 10.2144/01316bt01. 11768655

[ref67] Gao L.; Smith R. T. Optical hyperspectral imaging in microscopy and spectroscopy - a review of data acquisition. Journal of Biophotonics 2015, 8 (6), 441–456. 10.1002/jbio.201400051. 25186815 PMC4348353

[ref68] Zimmermann T. Spectral imaging and linear unmixing in light microscopy. In Microscopy Techniques; Rietdorf J., Ed.; Advances in Biochemical Engineering-Biotechnology, Vol. 95; 2005; pp 245–265. 10.1007/b102216 16080271

[ref69] Battaile C. C. The kinetic Monte Carlo method: Foundation, implementation, and application. Computer Methods in Applied Mechanics and Engineering 2008, 197 (41–42), 3386–3398. 10.1016/j.cma.2008.03.010.

[ref70] Voter A. F. Introduction to the Kinetic Monte Carlo Method. In Conference of the NATO-Advanced-Study-Institute on Radiation Effects in Solids, Erice, ITALY, Jul 17–29, 2004; Vol. 235, pp 1–23.

