# Supplementary material for: Transition from Light-Induced Phase Reconstruction to Halide Segregation in CsPbBr3–xIx Nanocrystal Thin Films
Source: ACS Appl Mater Interfaces. 2025 Feb 20;17(9):14389–403. doi: 10.1021/acsami.4c19234 (PMC11891859; doi:10.1021/acsami.4c19234)
Supplement: Supplementary file 1 — am4c19234_si_001.pdf [file am4c19234_si_001.pdf]

SUPPORTING INFORMATION

Transition from Light-Induced Phase Reconstruction to Halide Segregation in $CsPbBr_{3-x}I_x$ Nanocrystal Thin Films

Thiago Rodrigues da Cunha[1], Diego Lourençoni Ferreira[1], Letícia Ferreira Magalhães[2], Thaís Adriany de Souza Carvalho[2], Gabriel Fabrício de Souza,[1] Jefferson Bettini[5], Angelo Danilo Faceto[3], Cleber Renato Mendonça[4], Leonardo de Boni[4], Marco Antônio Schiavon[2], Marcelo Gonçalves Vivas[1]*

[1] Laboratório de Espectroscopia Óptica e Fotônica, Universidade Federal de Alfenas, 37715-400 Poços de Caldas, MG, Brazil

[2]Grupo de Pesquisa em Química de Materiais, Universidade Federal de São João del-Rei, 36301-160 São João del-Rei, MG, Brazil

[3]Universidade Federal dos Vales do Jequitinhonha e Mucuri - Campus JK, Instituto de Ciências Agrárias

[4]Instituto de Física de São Carlos, Universidade de São Paulo, São Carlos, SP 13566-590, Brazil

[5]Laboratório Nacional de Nanotecnologia, Centro Nacional de Pesquisa em Energia e Materiais, 13083-970 Campinas, São Paulo, Brazil

*Corresponding author: *mavivas82@gmail.com*

1 – Transmission electron microscopy images

Figure S1 illustrates the TEM images and corresponding nanoparticle (NP) count histograms for the synthesized colloidal perovskite nanocrystals.

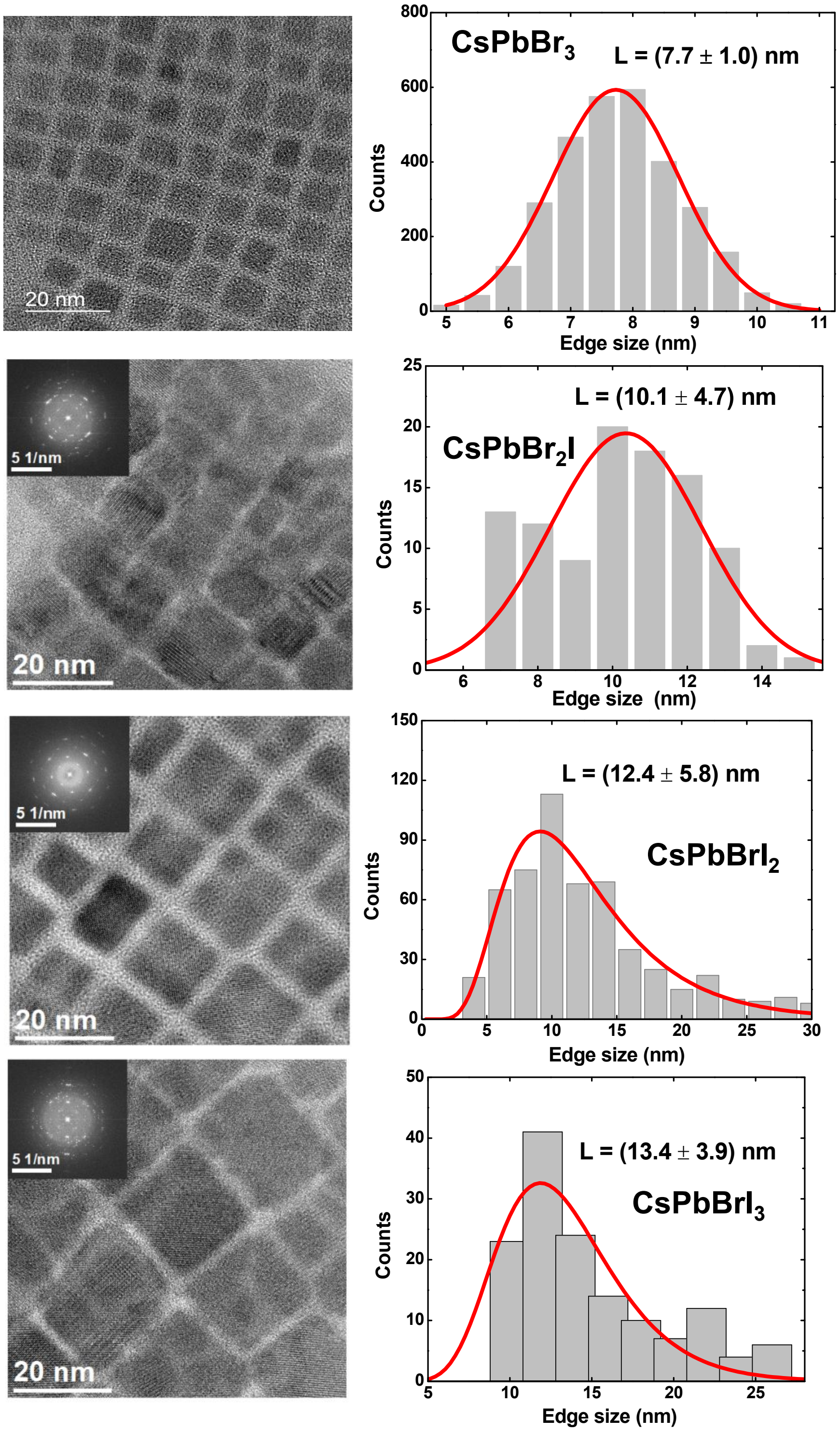

Figure S1 – TEM images and related NP count histograms for colloidal perovskite nanocrystals.

2 – Fluorescence PR rates

Table S1: Fluorescence average PR rates for the $CsPbBr_2I$ and $CsPbBr_{1.5}I_{1.5}$ nanocrystal thin films.

**$CsPbBr_2I$ nanocrystal thin films**					
P (μW)	$<k_{01}>$	$<k_1>$	$<k_{02}>$	$<k_2>$	$<k>$
5	0.62038	0.00113	0.31473	0.000975	0.00108
10	0.65902	0.00157	0.31745	0.00217	0.00176
20	0.64908	0.00203	0.31736	0.00203	0.00203
30	0.66305	0.00325	0.3245	0.00478	0.00376
40	0.67681	0.00532	0.3945	0.00553	0.00539
50	0.55722	0.00361	0.41301	0.00661	0.00489
60	0.87896	0.01168	0.15916	0.00278	0.01032
70	0.98089	0.00852	0.031749	0.00726	0.00848
85	0.6381	0.00957	0.35351	0.00739	0.00879
**$CsPbBr_{1.5}I_{1.5}$ nanocrystal thin films**					
P (μW)	$<k_{01}>$	$<k_1>$	$<k_{02}>$	$<k_2>$	$<k>$
5	0.0013	0.00713	0.99897	0.00102	0.00103
10	0.0015	0.00883	0.993664	0.00121	0.00122
20	0.33094	0.00748	0.70511	0.0001090	0.00246
40	0.40741	0.0088	0.62779	0.000304	0.00365
50	0.28839	0.01446	0.75664	0.0004875	0.00434
80	0.37544	0.01359	0.63322	0.00057	0.00533
100	0.26203	0.02055	0.80801	0.00118	0.00593
120	0.69384	0.00757	0.26776	0.0005050	0.00546
160	0.3253	0.01171	0.63637	0.00174	0.00512

3 – X-Ray Diffraction

Figure S2 exhibits the X-ray diffraction (XRD) patterns for $CsPbBr_{3-x}I_x$ (x = 0.0; 1.0; 1.5; 2.0; 3.0) nanocrystal thin films. The results are compatible with the cubic crystal system, Pm-3m space group (nº 221), reported by Carlos A. López *et. al.* in the Inorganic Crystal Structure Database (ICSD), nº 14610,[1] derived from high-resolution Synchrotron X-Ray Diffraction (SXRD) analysis of bulk polycrystalline $CsPbBr_3$ samples. Moreover, it is possible to verify a preferential growth orientation in the (100) family of planes ($2\theta \sim 15.08^{\circ}$), as can be seen in Figure S2 (a). Our measurements were performed using a conventional diffractometer (λ = 1.5418 Å). The Rietveld refinement was employed from the EXPGUI-GSAS software [2-5], which provided the lattice parameter and volume of the cubic unit cell, as can be seen in Figure S2 (b). An almost linear expansion of the lattice is observed due to the replacement of Br^- by I^-.

We also have estimated the crystallite size L for the nanocrystals deposited on a substrate through the Debye-Scherrer equation :

$$L = 0.9\frac{1.54\times10^{-10}}{\beta\cos(\theta)}, \quad (1)$$

in which 0.9 represents the Scherrer constant, the numerical factor 1.54×10^{-10} is the Cu Kα irradiation wavelength, β (radians) is the full-width at half-maximum (FWHM) of the diffraction peaks, θ (radians) is the Bragg's diffraction angle. In order to calculate the crystallite size L, we made an average on all diffraction peaks observed in the diffractogram. Table S1 illustrates the average edge length of the cube-shaped NPs obtained from TEM and XRD measurements. As can be noted, the NP size tends to increase as a function of the iodine content.

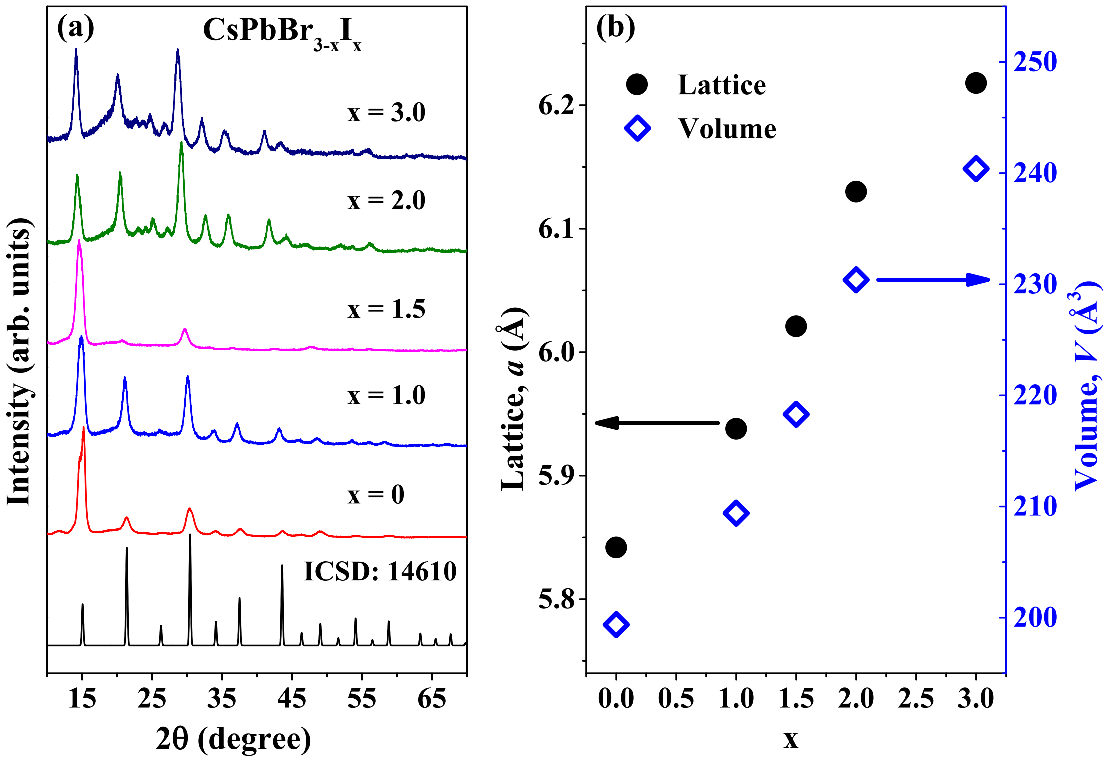

Figure S2 – (a) Diffraction patterns obtained for the perovskite nanocrystal thin films of $CsPbBr_{3-x}I_x$ (0 < x < 3), (b) lattice parameter and unit cell volume as functions of the I/Br concentration (x). The diffraction peaks were indexed using the crystallographic records of the Inorganic Crystal Structure Database (ICSD), nº 14610.

Table S2 – Average edge length (L) obtained from the TEM and XRD measurements.

	L_{TEM} (nm)	L_{XRD} (nm)
$CsPbBr_3$	7.7 ± 1.0	7.6 ± 1.3
$CsPbBr_2I$	10.1 ± 4.7	9.7 ± 2.0
$CsPbBr_{1.5}I_{1.5}$	-	7.5 ± 1.0
$CsPbBrI_2$	12.4 ± 5.8	10.0 ± 1.4
$CsPbI_3$	13.4 ± 3.9	11.0 ± 1.9

4 – Fluorescence lifetime

The time-resolved fluorescence curves are depicted in Figure S3, while the corresponding fitting parameters are shown in Table S2.

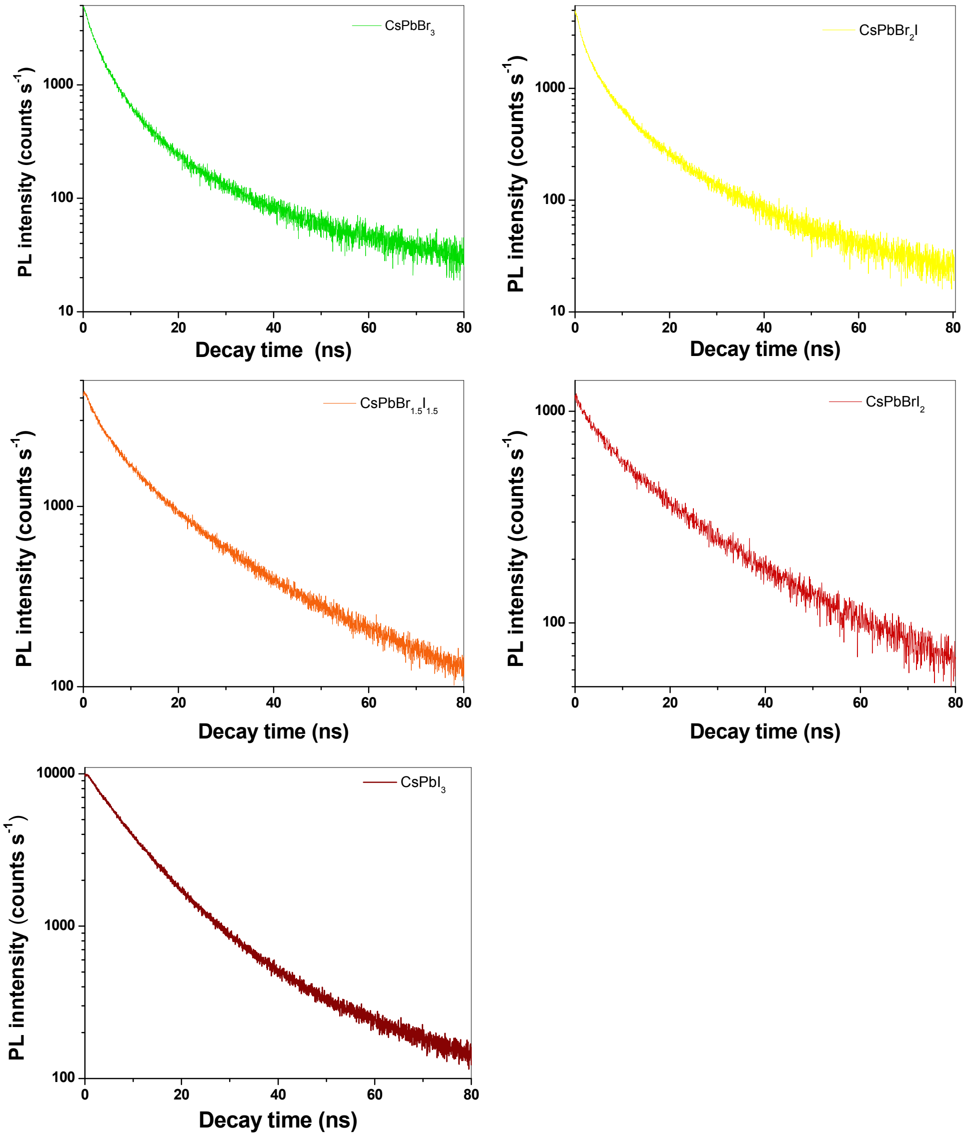

Figure S3 – Fluorescence decay curves obtained for the perovskite nanocrystal thin films.

Table S3 – Fitting parameters obtained from the fluorescence decay curves for the perovskite nanocrystal thin films.

	**A_1 (%)**	**τ_1 (ns)**	**A_2 (%)**	**τ_2 (ns)**	**τ_{av} (ns)**
$CsPbBr_3$	28.45	2.32	71.55	8.13	7.54
$CsPbBr_2I$	93.21	10.47	6.79	1.75	10.37
$CsPbBr_{1.5}I_{1.5}$	89.97	15.05	10.03	3.2	14.77
$CsPbBrI_2$	95.88	15.62	4.12	2.55	15.53
$CsPbI_3$	54.03	18.12	45.97	7.72	15.35

5 – Determination of laser-induced temperature

We applied the finite-difference method to evaluate the temperature induced by the laser in the spin-coated nanocrystalline $CsPbBr_{3-x}I_x$ perovskite film. [6, 7] During continuous-wave laser excitation, the efficient conversion of the energy from the laser beam, absorbed by the lattice electrons of the material, into heat allows us to ascertain the local temperature (T) at any given time (t) across the two spatial dimensions (x, y) of the irradiated thin film, as outlined by the classical Fourier heat equation: [8]

$$\rho c_p \frac{\partial T(x,y,t)}{\partial t} - \frac{\partial}{\partial y}\left[K\frac{\partial T(x,y,t)}{\partial y}\right] - \frac{\partial}{\partial x}\left[K\frac{\partial T(x,y,t)}{\partial x}\right] = Q(x,y,t)\,, \tag{1}$$

In this equation, ρ = 4730 kg/m^3 [9, 10] represents the average density of the nanocrystals, cp = 300 J/(kg·K)[11] is the specific heat capacity, and K = 0.43 W/(m·K) [11, 12] denotes the thermal conductivity of polycrystalline $CsPbBr_3$. Additionally, the volumetric heat source created by the laser beam hitting the top surface of the material is described by:

$$Q(x,y,t) = \beta(1-R-\varphi)I(x,y)e^{-\beta L}\,, \tag{2}$$

in which R is the reflectance, φ is the fluorescence quantum yield of the thin films ($\varphi_{CsPbBr_2I} = 0.5$, $\varphi_{CsPbBr_2I} = 0.4$ and $\varphi_{CsPbBr_2I} = 0.3$), β (405 nm) is the absorption coefficient (2-3 x 10^6 m^{-1}),[13-15] and L (~40-60 nm) is the thin film thickness. Given that our laser beam has a Gaussian intensity profile, we employed the following equation:

$$I(x,y) = \frac{2\overline{P}}{\pi w_0^2} e^{-\left[\frac{2(x-x_0)^2}{w_0^2} + \frac{2(y-y_0)^2}{w_0^2}\right]}\,. \tag{3}$$

In this expression, I(x,y) refers to the peak intensity (W/m²) at the spatial coordinates (x,y), $\overline{P}$ is the average laser power (W), w_0 represents the laser waist radius (m), estimated at 1 μm using the zero-damage method and x_0 and y_0 indicate the grid points where the laser is incident. Initially, we assumed the perovskite thin film was at room temperature ($T(x,y,t_0) = 293$ K) at the start of laser irradiation. Convection losses were not considered due to their minor impact in thin films, and radiation losses were ignored because of the high laser intensity used (10^6 W/m²). We also assumed that the intrinsic properties of the perovskite material, including thermal conductivity, density, and heat capacity, remained unchanged despite variations in temperature. Within the finite-difference method framework, the Thomas Algorithm was utilized to solve the system of coupled equations and ensure the stability condition $\alpha dt/((dx)^2) < 1/2$, where α = K/(ρc□) is the thermal diffusivity. The computational model was implemented using Python programming language.

6 – Hyperspectral fluorescence microscopy

Figure S4 illustrates a schematic representation of the optical arrangement used in our hyperspectral fluorescence microscopy experiments. A continuous-wave (cw) laser emits light at 405 nm, which is directed through a spatial filter to achieve the TEM_{00} mode. Subsequently, a telescope collimates and expands the laser beam, covering the entire entrance of the microscope objective and creating a focal beam waist near the diffraction limit, thereby maximizing the peak intensity. The 405 nm beam passes through a half-wave plate and a calcite polarizer to control incident power without altering the polarization state or beam position.

The laser beam is then directed downward by a 405 nm dielectric mirror toward the microscope objective. This objective lens possesses a 40x magnification, a numerical aperture of 0.65, and a working distance of 0.6 mm. As the light beam is focused on the sample (the beam waist radius w_0 = 1 μm was obtained from the zero-damage method) [23] put on the XYZ translation stage (with a resolution of <1 μm), it is absorbed, leading to fluorescence emission. The objective lens captures this fluorescence, initiating the reverse optical path. The collected fluorescence is directed to the dielectric mirror, which allows fluorescence transmission while reflecting the laser excitation. Subsequently, the fluorescence passes through a 405 nm filter and converging lens, focusing the beam onto an optical fiber connected to a portable spectrometer.

The entire optical system is controlled by dedicated software. All measurements were performed at room temperature (293 K) and in an air-saturable atmosphere.

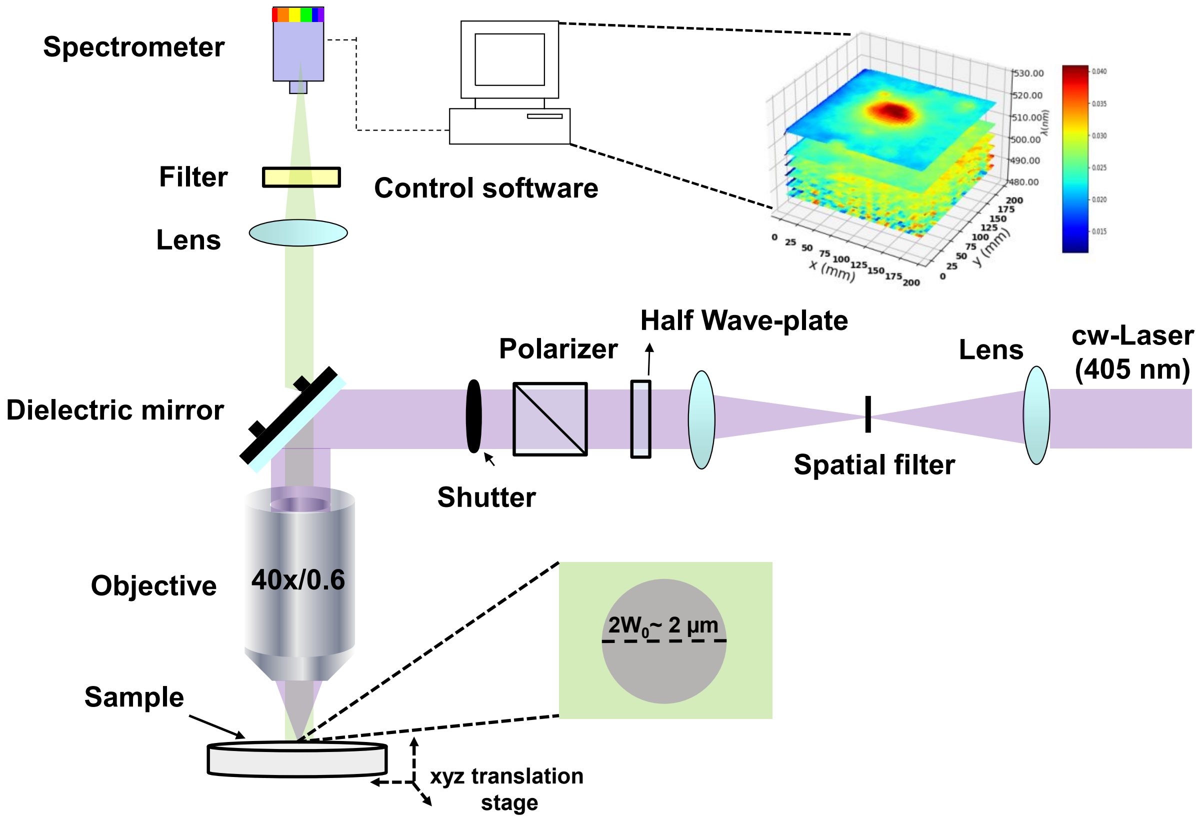

Figure S4 – Hyperspectral fluorescence microscopy setup.

References

(1) López, C. A.; Abia, C.; Alvarez-Galván, M. C.; Hong, B. K.; Martínez-Huerta, M. V.; Serrano-Sánchez, F.; Carrascoso, F.; Castellanos-Gómez, A.; Fernández-Díaz, M. T.; Alonso, J. A. Crystal Structure Features of CsPbBr₃ Perovskite Prepared by Mechanochemical Synthesis. *Acs Omega* **2020**, *5* (11), 5931-5938. DOI: 10.1021/acsomega.9b04248.

(2) *2004*; Los Alamos National Laboratory Report LAUR 86-748, (accessed.

(3) McCusker, L. B.; Von Dreele, R. B.; Cox, D. E.; Louër, D.; Scardi, P. Rietveld refinement guidelines. *Journal of Applied Crystallography* **1999**, *32*, 36-50. DOI: 10.1107/s0021889898009856.

(4) VonDreele, R. B. Quantitative texture analysis by Rietveld refinement. *Journal of Applied Crystallography* **1997**, *30*, 517-525. DOI: 10.1107/s0021889897005918.

(5) Rietveld, H. M. LINE PROFILES OF NEUTRON POWDER-DIFFRACTION PEAKS FOR STRUCTURE REFINEMENT. *Acta Crystallographica* **1967**, *22*, 151-&. DOI: 10.1107/s0365110x67000234.

(6) Recktenwald, G. W. *Numerical Methods with MATLAB: Implementations and Applications*; 2000.

(7) Recktenwald, G. W. Finite-Difference Approximations to the Heat Equation. 2004.

(8) Williams, E.; Brousseau, E. B. Simulation and Experimental Study of Nanosecond Laser Micromachining of Commercially Pure Titanium. *J. Micro Nano-Manuf.* **2016**, *4*, 011004.

(9) Skurlov, I. D.; Yin, W. X.; Ismagilov, A. O.; Tcypkin, A. N.; Hua, H. H.; Wang, H. B.; Zhang, X. Y.; Litvin, A. P.; Zheng, W. T. Improved One- and Multiple-Photon Excited Photoluminescence from Cd²⁺-Doped CsPbBr₃ Perovskite NCs. *Nanomaterials* **2022**, *12* (1). DOI: 10.3390/nano12010151.

(10) *https://next-gen.materialsproject.org/materials/mp-600089*.

(11) Haeger, T.; Heiderhoff, R.; Riedl, T. Thermal properties of metal-halide perovskites. *Journal of Materials Chemistry C* **2020**, *8* (41), 14289-14311. DOI: 10.1039/d0tc03754k.

(12) Lee, W.; Li, H. S.; Wong, A. B.; Zhang, D. D.; Lai, M. L.; Yu, Y.; Kong, Q.; Lin, E.; Urban, J. J.; Grossman, J. C.; et al. Ultralow thermal conductivity in all-inorganic halide perovskites. *Proceedings of the National Academy of Sciences of the United States of America* **2017**, *114* (33), 8693-8697. DOI: 10.1073/pnas.1711744114.

(13) Maes, J.; Balcaen, L.; Drijvers, E.; Zhao, Q.; De Roo, J.; Vantomme, A.; Vanhaecke, F.; Geiregat, P.; Hens, Z. Light Absorption Coefficient of CsPbBr₃ Perovskite Nanocrystals. *Journal of Physical Chemistry Letters* **2018**, *9* (11), 3093-3097. DOI: 10.1021/acs.jpclett.8b01065.

(14) Mannar, S.; Mandal, P.; Roy, A.; Viswanatha, R. Experimental Determination of the Molar Absorption Coefficient of Cesium Lead Halide Perovskite Quantum Dots. *Journal of Physical Chemistry Letters* **2022**, *13* (27), 6290-6297. DOI: 10.1021/acs.jpclett.2c01198.

(15) Huang, Y.; Zhang, L. C.; Wang, J. B.; Zhang, B. Y.; Xin, L. J.; Niu, S. R.; Zhao, Y.; Xu, M.; Chu, X. B.; Zhang, D. Y.; et al. Growth and optoelectronic application of CsPbBr₃ thin films deposited by pulsed-laser deposition. *Optics Letters* **2019**, *44* (8), 1908-1911. DOI: 10.1364/ol.44.001908.
